# Boosting distributional copula regression for bivariate binary, discrete and mixed responses

**DOI:** 10.1177/09622802241313294

**Published:** 2025-03-21

**Authors:** Guillermo Briseño Sanchez, Nadja Klein, Hannah Klinkhammer, Andreas Mayr

**Affiliations:** 1 Methods for Big Data, Scientific Computing Center, 150232 Karlsruhe Institute of Technology, Karlsruhe, Germany; 2 Department of Medical Biometrics, Informatics and Epidemiology, Faculty of Medicine, University of Bonn, Bonn, Germany

**Keywords:** Dependence modelling, GAMLSS, model-based boosting, shrinkage, variable selection

## Abstract

Motivated by challenges in the analysis of biomedical data and observational studies, we develop statistical boosting for the general class of bivariate distributional copula regression with arbitrary marginal distributions, which is suited for binary, count, continuous or mixed outcomes. To arrive at a flexible model for the entire conditional distribution, not only the marginal distribution parameters but also the copula parameters are related to covariates through additive predictors. We suggest estimation by means of an adapted component-wise gradient boosting algorithm. A key benefit of boosting as opposed to classical likelihood or Bayesian estimation is the implicit data-driven variable selection mechanism as well as shrinkage. To the best of our knowledge, our implementation is the only one that combines a wide range of covariate effects, marginal distributions, copula functions, and implicit data-driven variable selection. We showcase the versatility of our approach to data from genetic epidemiology, healthcare utilization and childhood undernutrition. Our developments are implemented in the R package gamboostLSS, fostering transparent and reproducible research.

## 1. Introduction

Distributional regression models have gained considerable prominence in statistical research over the last decade, thereby moving the focus from modelling the conditional mean of the response variable (as done in classical regression) towards modelling the entire conditional distribution.^1^ Such models capable of describing the complete distribution are highly relevant in biomedical research, as they allow to explore variables that impact not only the average value of biomarkers, phenotypes or scores but also other quantities such as variance or quantiles. Common examples are the construction of reference curves or growth charts, where skewness is often covariate-specific^2,3^; or bivariate time-to-event data.^4^

Several distinct approaches to distributional regression for univariate responses exist, see Klein^1^ for a recent review. Our framework builds on generalized additive models for location, scale shape (GAMLSS),^5^ which allow us to relate all distribution parameters of an arbitrary univariate parametric distribution to covariates. A simple example is a GAMLSS for the Gaussian regression model, in which not only the expectation, but also the standard deviation can be related to covariates. This allows, for example, model heteroscedasticity. While originally proposed for univariate responses, GAMLSS has been extended to accommodate regression models for multivariate responses,^6^ although practically most existing approaches are limited to the bivariate case.^7–9^ While parametric bivariate distributions such as the bivariate Gaussian, bivariate Bernoulli or bivariate Poisson offer an avenue for modelling bivariate responses, they also impose limitations on the distribution of the margins, for example, being univariate Gaussian or Poisson. A flexible alternative way to construct bivariate distributions is copulas.^10^ This approach allows to linking of arbitrary marginal distributions through a copula function, reflecting the association between the components. The literature on copula modelling, including the regression setting, is vast see, for example , Smith^11^ for a review.

Reflecting the diversity of response types in our biomedical applications, in this paper, we are particularly concerned with situations where the response variable is a bivariate vector $Y=(Y_{1},Y_{2}{)}^{⊤}$ with dependent components on possibly different domains. We construct bivariate distributions for such situations via conditional copulas and parametric margins; and allow all distributional parameters of the joint density to depend on covariates. Estimation is realized jointly rather than employing a two-step procedure frequently employed in copula models. Recent contributions that are akin to ours can be found in Marra and Radice^12^ featuring a bivariate continuous response, Marra and Radice^13^ using bivariate binary outcomes, van der Wurp et al.^14^ studying bivariate count responses, as well as Klein et al.^15^ analysing a mixed binary and continuous response. All these contributions showed how to construct highly flexible bivariate copula regression models that are able to accommodate a wide range of covariate effects as well as response types. However, the substantial flexibility inherent in this model class of distributional copula regression models notably exacerbates the issue of variable selection—a challenge that currently remains unaddressed within the specific models we are considering.

**Figure 1.**
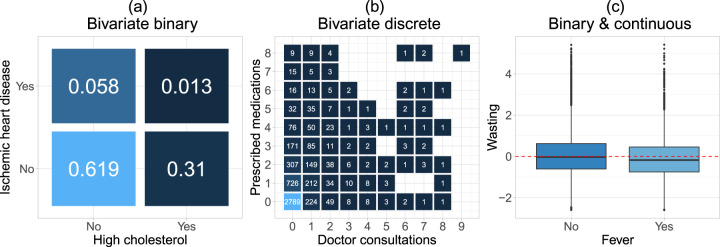
Responses in our applications analysed in Section 4: (a) binary–binary response (numbers indicate proportions): high cholesterol and chronic ischemic heart disease; (b) count–count response (numbers indicate cases): doctor visits and medical prescriptions; and (c) binary–continuous response: fever and wasting (indicator for acute undernutrition).

Our methodological contribution builds on the recent work by Hans et al.,^16^ who estimated bivariate distributional copula regression models via a component-wise gradient boosting framework. However, in this approach, the response variables are both required to be strictly continuous. In many biomedical applications (but not only there), data are often recorded at a discretized scale (e.g. symptoms present yes/no) or the responses of interest actually depict a phenomenon expressed through discrete numbers/positive integers as in, for example, the number of doctor appointments or the number of prescription medications designated to a patient. At the time of writing (December 2023), a search in PubMed (https://pubmed.ncbi.nlm.nih.gov) returns 395,078 and 24,439 results for “logistic regression” and “Poisson regression” since 2010, respectively, highlighting the prevalence of binary and count responses. It may also be the case that the biomedical outcome is expressed as a combination of responses that lie in different domains, for example, a binary indicator and a continuous measurement reflecting a disease (or symptom) indicator and an undernutrition score. The three aforementioned types of responses are the ones we consider later in Section 4 and the marginal distributions are visualized in Figure 1.

Recent work by Strömer et al.^17^ combined multivariate distributional regression with gradient boosting in order to fit interpretable and highly flexible regression models in high-dimensional biomedical settings for bivariate continuous, bivariate binary and bivariate count responses. Their work considered the bivariate Poisson and the bivariate Bernoulli distributions, which suffer from some limitations. On the one hand, the bivariate Poisson distribution is only able to model positive association structures between the margins. On the other hand, the bivariate Bernoulli distribution models the association between the marginal responses by means of the ‘odds ratio’, whose ease of interpretation remains at best questioned, see, for example, Norton et al.^18^ Furthermore, the marginal distributions of the components in the response vector are assumed to be of the same type, that is, the margins of a bivariate Bernoulli/Poisson distribution must be univariate Bernoulli/Poisson distributions. Such a restrictive assumption might not always be supported by the data. For example, in one of our applications where we study childhood undernutrition via the joint distribution of *wasting*, a continuous indicator for acute undernutrition as reflected by low weight-for-height (in comparison to a reference population), and a binary indicator for fever within the two weeks preceding a survey interview.

The aim of this article is threefold: First, we build upon Hans et al.^16^ and extend the class of boosting bivariate distributional copula regression models to arbitrary margins on different domains. Second, we expand the catalogue of copula functions and families of marginal distributions available for the publicly available R^19^ package gamboostLSS.^20^ These new additions allow for conducting data-driven variable selection and shrinkage in both low- and high-dimensional applications, where the number of candidate variables ( p ) may greatly exceed the number of observations ( n ). Third, we demonstrate the versatility and wide applicability of our approach through three biomedical applications.

The rest of the article is structured as follows: Section 2 reviews distributional copula regression with different types of responses and outlines our boosting algorithm. Section 3 summarizes our simulation studies as well as their respective results. Section 4 presents the three case studies where we analyse data from epidemiological applications in genetic epidemiology, healthcare and public health in developing countries. We additionally illustrate the model-building process that involves selecting marginal distributions as well as copula distributions. Lastly, a discussion is given in Section 5. Supplemental material C contains further details on the simulation studies.

## 2. Bivariate distributional copula regression

### 2.1. Model structure

We assume that the i th observation of a response, with i=1,…,n , follows a parametric distribution. In the context of bivariate responses considered here, the joint distribution of the random vector $Y=(Y_{i1},Y_{i2}{)}^{⊤}$ is denoted by $P(Y_{1}\leq y_{i1},\;Y_{2}\leq y_{i2};\vartheta_{i})=F_{1,2}(y_{i1},y_{i2};\vartheta_{i}),$ where $F_{1,2}(\cdot ;\vartheta_{i})$ represents the joint cumulative distribution function (CDF) parameterized through a K -dimensional parameter vector $\vartheta_{i}=(\vartheta_{i1},\ldots ,\vartheta_{iK}{)}^{⊤}$ . Rather than assuming a joint parametric distribution for Y , we resort to a copula-based approach using Sklar’s theorem.^10^ This theorem states that any bivariate distribution can be written as

(1) $$F(y_{i1},y_{i2};\vartheta_{i})=C[F_{1}(y_{i1};\vartheta_{i}^{\left(1\right)}),F_{2}(y_{i2};\vartheta_{i}^{\left(2\right)});\vartheta_{i}^{\left(c\right)}]$$

where $C(\cdot ,\cdot ):[0,1{]}^{2}\to [0,1]$ is the CDF of a bivariate parametric copula function with parameters $\vartheta_{i}^{\left(c\right)}\in R^{K_{c}}$ . The copula *links* the possibly different parametric marginal distributions with CDFs $F_{1},F_{2}$ and respective parameter vectors $\vartheta_{i}^{\left(1\right)}\in R^{K_{1}}$ , $\vartheta_{i}^{\left(2\right)}\in R^{K_{2}}$ to arrive at the joint bivariate distribution. In what follows, we consider one-parametric bivariate copulas and refer to $\vartheta_{i}^{\left(c\right)}=\vartheta_{i}^{\left(c\right)}$ as the corresponding scalar association parameter that determines the strength of the association between the marginal responses. Table SA1 in Supplemental material A details the implemented copulas in the R add-on package gamboostLSS.

Let now $K=K_{1}+K_{2}+K_{c}=K_{1}+K_{2}+1$ denote the total number of distribution parameters in the bivariate distribution and $\vartheta_{i}^{\left(1\right)}=(\vartheta_{i1}^{\left(1\right)},\ldots ,\vartheta_{iK_{1}}^{\left(1\right)}{)}^{⊤}$ , $\vartheta_{i}^{\left(2\right)}=(\vartheta_{i1}^{\left(2\right)},\ldots ,\vartheta_{iK_{2}}^{\left(2\right)}{)}^{⊤}$ , be the vectors containing all parameters that correspond to the respective marginal distributions. All K parameters of the bivariate distribution are then stored in the vector $\vartheta_{i}=((\vartheta_{i}^{\left(1\right)}{)}^{⊤},(\vartheta_{i}^{\left(2\right)}{)}^{⊤},\vartheta_{i}^{\left(c\right)}{)}^{⊤}$ . The distributional copula regression approach relates each component of $\vartheta_{i}$ to possibly different subvectors of the covariate information $x_{i}$ . More precisely, we employ structured additive predictors of the form

(2) $$g_{k}^{\left(∙\right)}(\vartheta_{ik}^{\left(∙\right)})=\eta_{ik}^{\left(∙\right)}=\beta_{0k}^{\left(∙\right)}+\sum_{r=1}^{P_{k}^{\left(∙\right)}}s_{rk}^{\left(∙\right)}(x_{ir})$$

where $g_{k}^{\left(∙\right)}(\cdot )$ are suitable link functions with corresponding inverse functions $h_{k}^{\left(∙\right)}(\cdot )$ that ensure potential parameter space restrictions. The symbol ∙∈{1,2,c} and the summation limit $P_{k}^{\left(∙\right)}$ emphasize that the individual parameters $\vartheta_{ik}^{\left(∙\right)}$ do not necessarily have to be modelled using the same subset of covariates. The coefficients $\beta_{0k}^{\left(∙\right)}$ are parameter-specific intercepts and $s_{rk}^{\left(∙\right)}(\cdot )$ are smooth functions that can accommodate a wide range of functional forms of the covariates, such as linear, non-linear, or spatial effects. Each covariate effect is modelled by appropriate basis function expansions of the form

$$s_{rk}^{\left(∙\right)}(x)=\sum_{l=1}^{L_{rk}^{\left(∙\right)}}\beta_{rk,l}^{\left(∙\right)}B_{rk,l}^{\left(∙\right)}(x)$$

where $B_{rk,l}^{\left(∙\right)}(x)$ is a suitable basis function evaluated at the observed covariate value and $\beta_{rk,l}^{\left(∙\right)}$ are generic coefficients to be estimated. As a final remark, Sklar’s theorem guarantees that the copula characterising the joint distribution of $Y_{i}$ is unique only if the marginal responses are continuous. When discrete margins are present, the copula is uniquely defined only on the range of the marginal CDFs. However, within our framework, identifiability should not be an issue for two reasons. First, we fix the parametric form of the joint distribution a priori by making choices for the marginal distributions and the copula function. Thereby, potential identifiability issues that arise when one is interested in learning, for example, the copula and the marginals in a nonparametric framework without an a priori fixed structure, are not present. Second, identifiability is ensured in our regression setting where all parameters of the distribution are observation-specific. For this, consider two observations in the sample, say i and $i^{\prime }$ , with the same observed marginal response ( $y_{ij}=y_{ij^{\prime }}$ ) but different covariate values for j=1,2 . Modelling the parameters of the respective marginal distributions as functions of covariates results in different estimates for the marginal CDFs, that is, ${\hat{F}}_{j}(y_{ij};{\hat{\vartheta }}_{i})\neq {\hat{F}}_{j}(y_{ij^{\prime }};{\hat{\vartheta }}_{i^{\prime }}),j=1,2$ . Hence a richer range of the estimated CDFs of the discrete marginal distributions is obtained, mitigating the identification issue when using these types of marginal responses. This has been also pointed out by other researchers.^21–24^

### 2.2. Relevant examples of bivariate responses

In the following, we briefly describe the bivariate responses relevant to our applications. The respective choices of corresponding marginal distributions are summarized in Table SA2 in Supplemental material A together with main characteristics, such as expectation and variance.

#### 2.2.1. Bivariate binary responses

We begin by considering the case $Y_{ij}\in \{0,1\}$ , j=1,2 . The individual marginal probabilities of observing $y_{ij}=1$ are modelled via $P(Y_{ij}=1;\vartheta_{i}^{\left(j\right)})=\vartheta_{i}^{\left(j\right)}=h^{\left(j\right)}(\eta_{i}^{\left(j\right)})=:p_{i}^{1(j)}$ , j=1,2 , where the response function can be any function suitable for parameters whose range is the unit interval [0,1] , for example, logit, probit and cloglog link functions. Then,

$$\begin{array}{rl} P(Y_{i1}=1,Y_{i2}=1;\vartheta_{i}) & =C[P(Y_{i1}=1;\vartheta_{i}^{\left(1\right)}),P(Y_{i2}=1;\vartheta_{i}^{\left(2\right)});\vartheta_{i}^{\left(c\right)}]\\ & =:p_{i}^{11} \end{array}$$

The joint probability mass function consists of the four possible outcomes of the binary responses, that is, $\left(y_{i1},y_{i2})\in \{(1,1),(1,0),(0,1),(0,0)\right\}$ . This leads to the following log-likelihood contribution of the i th observation.

(3) $$\begin{array}{rl} \ell_{i} & =y_{i1}y_{i2}\log\;(p_{i}^{11})+y_{i1}(1-y_{i2})\log\;(p_{i}^{1(1)}-p_{i}^{11})\\ & \;+(1-y_{i1})y_{i2}\log\;(p_{i}^{1(2)}-p_{i}^{11})\\ & \;+(1-y_{i1})(1-y_{i2})\log\;(1-p_{i}^{1(1)}-p_{i}^{1(2)}+p_{i}^{11}) \end{array}$$

Note that our implementation allows the individual marginal probabilities to be modelled using different link functions.

#### 2.2.2. Bivariate discrete responses

Each marginal response is a count variable, that is, $Y_{ij}\in N_{\geq 0}$ , j=1,2 . Here we denote with $P(Y_{ij}\leq y_{ij};\vartheta_{i}^{\left(j\right)})=F_{j}(y_{ij};\vartheta_{i}^{\left(j\right)})$ the marginal CDFs, and with $P(Y_{ij}=y_{ij};\vartheta_{i}^{\left(j\right)})=f_{j}(y_{ij};\vartheta_{i}^{\left(j\right)})$ the marginal probability distribution functions of $Y_{ij}$ . Similar to van der Wurp et al.,^14^ we compute $P(Y_{ij}=y_{ij}-1;\vartheta_{i}^{\left(j\right)})=F_{j}(y_{ij};\vartheta_{i}^{\left(j\right)})-f_{j}(y_{ij};\vartheta_{i}^{\left(j\right)})$ in order to avoid a (trivial) evaluation of the CDF of $Y_{ij}$ with a negative argument in case that $y_{ij}=0$ , j=1,2 . The log-likelihood function of the i th observation is then given by

(4) ℓi=log{C[F1(yi1;ϑi(1)),F2(yi2;ϑi(2));ϑi(c)]−=C[F1(yi1;ϑi(1))−f1(yi1;ϑi(1)),F2(yi2;ϑi(2);ϑi(c))]−=C[F1(yi1;ϑi(1)),F2(yi2;ϑi(2))−f2(yi2;ϑi(2));ϑi(c)]+=C[F1(yi1;ϑi(1))−f1(yi1;ϑi(1)),F2(yi2;ϑi(2))−f2(yi2;ϑi(2));ϑi(c)]}

We have implemented various discrete distributions, including the Poisson and Geometric distributions. Additionally, we have integrated two-parameter count distributions designed for over-dispersed data such as the negative binomial (Type I). To handle count data characterized by an excess of zero observations, we have included zero-inflated and zero-altered distributions. These include models such as the zero-altered logarithmic, zero-altered negative binomial, zero-inflated Poisson and zero-inflated negative binomial distributions, see Table SA2 in Supplemental material A for a detailed description as well as Rigby et al.

#### 2.2.3. Bivariate mixed binary–continuous responses

When one response component is continuous and the other binary, we follow Klein et al.^15^ and resort to a latent variable representation of the regression model for the binary component. Without loss of generality, let the first component of the bivariate vector be the binary variable, that is, $Y_{i1}\in \{0,1\}$ . The binary response $Y_{i1}$ is then determined by an unobserved, latent variable $Y_{i1}^{*}$ with parametric CDF $F_{1}^{*}(y_{i1}^{*};\vartheta_{i}^{\left(1\right)})$ through the mechanism: $Y_{i1}=1(Y_{i1}^{*}>0)$ , where 1(⋅) is the indicator function. Then it follows that $P(Y_{i1}=0;\vartheta_{i}^{\left(1\right)})=F_{1}(0;\vartheta_{i}^{\left(1\right)})=F_{1}^{*}(0;\vartheta_{i}^{\left(1\right)})=P(Y_{i1}^{*}\leq 0;\vartheta_{i}^{\left(1\right)})$ , in other words, the CDFs of the binary and latent variables coincide at $y_{i1}=y_{i1}^{*}=0$ . With this representation, the joint bivariate distribution is

$$\begin{array}{rl} P(Y_{i1}=0,Y_{i2}\leq y_{i2};\vartheta_{i}) & =P(Y_{i1}^{*}\leq 0,Y_{i2}\leq y_{i2})\\ & =C[F_{1}^{*}(0;\vartheta_{i}^{\left(1\right)}),F_{2}(y_{i2};\vartheta_{i}^{\left(2\right)});\;\vartheta_{i}^{\left(c\right)}] \end{array}$$

from which we obtain the log-likelihood contribution:

(5) $$\begin{array}{rl} \ell_{i} & =(1-y_{i1})\log\;\left\{\frac{\partial C[F_{1}(0;\vartheta_{i}^{\left(1\right)}),F_{2}(y_{i2};\vartheta_{i}^{\left(2\right)});\vartheta_{i}^{\left(c\right)}]}{\partial F_{2}(y_{i2};\vartheta_{i}^{\left(2\right)})}\right\}\\ & \;+y_{i1}\log\;\left\{1-\frac{\partial C[F_{1}(0;\vartheta_{i}^{\left(1\right)}),F_{2}(y_{i2};\vartheta_{i}^{\left(2\right)});\vartheta_{i}^{\left(c\right)}]}{\partial F_{2}(y_{i2};\vartheta_{i}^{\left(2\right)})}\right\}+\log\;\left\{f_{2}(y_{i2};\vartheta_{i}^{\left(2\right)})\right\} \end{array}$$

The link function for the binary margin can be set to logit, probit, or cloglog.

### 2.3. Estimation via component-wise gradient boosting

As mentioned earlier and further emphasized by the summation index $P_{k}^{\left(∙\right)}$ shown in equation (2), there may not be strong a priori evidence of which subset of covariates (or if any at all) affects the individual parameters $\vartheta_{k}^{\left(∙\right)}$ of the bivariate distribution F(⋅,⋅;ϑ) . Therefore, we resort to component-wise gradient boosting or statistical boosting^25,26^ to estimate all coefficients simultaneously. While boosting is a general concept from machine learning, it has also been extended towards estimating statistical models.^27^ The term *component-wise* highlights that this particular boosting framework fits the base-learners (components) one-by-one and greedily updates the model by updating only the best-performing component.^28^ In our case, the base-learners are the additive components $s_{rk}^{\left(∙\right)}(x)$ in equation (2). We refer to Hothorn et al.^28^ and Mayr et al.^29^ for a complete list of the currently implemented base-learners. Estimating the model coefficients corresponds to solving the optimization problem:

$$\hat{\eta }={\arg\;\min}_{\eta }[E_{Y}\left\{\omega \left(Y;\eta \right)\right\}]$$

where the vector $\eta =(\eta^{\left(1\right)},\eta^{\left(2\right)},\eta^{\left(c\right)}{)}^{⊤}\in R^{K}$ contains all additive predictors corresponding to the parameters of the bivariate distribution and $\hat{\eta }$ denotes their estimates. The term ω(⋅) represents the loss function, which in our case corresponds to the negative log-likelihood of the regression model, that is, ω(⋅)=−ℓ(⋅) . In general, minimizing the expectation of the loss is intractable. In practice, given a sample of i=1,…,n observations, one minimizes the *empirical risk* $\rho =(1/n)\sum_{i=1}^{n}\omega (y_{i};\eta_{i})$ iteratively. In each boosting iteration, the algorithm fits each of the pre-specified base-learners in each predictor individually to the negative gradient of the loss function (also sometimes referred to as *pseudo-residuals)*, that is, $-\partial \rho /\partial \eta_{k}^{\left(∙\right)}$ . Only the best-fitting base-learner is selected and a ‘weak’ update of the model is conducted. The fitting procedure is run for a pre-specified number of iterations denoted by ${\text{m}}_{\text{stop}}$ , which plays a similar role like the penalty parameter ‘ λ ’ of the least absolute shrinkage and selection operator (LASSO),^30^ and acts as the main tuning parameter. In our case, we conduct non-cyclical updates,^31^ which means that only one out of all additive predictors is updated per fitting iteration. Only the update which leads to the highest decrease in the empirical risk is updated. By conducting *early stopping*, that is, using ${\text{m}}_{\text{stop}}^{\text{opt}}<{\text{m}}_{\text{stop}}$ fitting iterations, some base-learners will effectively be left out of the model, since they were not selected in any iteration. Hence early stopping results in intrinsic, data-driven variable selection as well as shrinkage of covariate effects. Algorithm 1 in Supplemental material A details the procedure including a mechanism for tuning of ${\text{m}}_{\text{stop}}$ .

The data-driven variable selection and regularization of effect estimates resulting from boosting with early stopping are particularly suitable for exploratory data analyses or prediction modelling. In such cases, boosting can provide valuable insights by automatically selecting relevant variables without requiring prior knowledge of their importance. However, it is worth noting that as a main limitation, statistical boosting in our flexible model class lacks the availability of asymptotic theory to, for example, construct confidence intervals or to conduct inference.

## 3. Simulation study

In this section, we summarize the main findings of our simulation study. We refer to Supplemental material B for all details of the simulation study. We consider three response scenarios in Sections SB1 to SB3 in Supplemental material B., one for bivariate binary, count and mixed outcomes each under different levels of sparsity. The main goals are to evaluate (i) estimation, (ii) variable selection and (iii) predictive performance of our proposed bivariate copula approach compared to the benchmark of estimating two separate (and thus independent) univariate models. Additionally, we investigate the performance of the out-of-sample negative log-likelihood evaluated on an additional test data set to identify the correct copula function in Section SB4 in Supplemental material B. The code used to reproduce the simulations can be found in the following repository: https://github.com/GuilleBriseno/BoostDistCopReg_BinDiscMix.

### 3.1. General settings

All boosting models are fitted using the gamboostLSS package. A training data set of $n_{\text{train}}=1000$ observations and a fixed step-length of ${\text{s}}_{\text{step}}=0.1$ for all distribution parameters are used. The stopping iteration ${\text{m}}_{\text{stop}}$ is optimized by minimising the out-of-bag negative log-likelihood using a validation data set with $n_{\text{mstop}}=1500$ observations from the same underlying distribution (see Step 4 in Algorithm 1 in Supplemental material A). We apply $L_{2}$ -stabilisation to the parameter-specific gradients in order to obtain similar step-lengths among the various dimensions of the model, see Hofner et al.^20^ for details on gradient stabilisation. The performance of the copula and univariate models is evaluated using multivariate proper scoring rules (negative log-likelihood and energy score^32^), both oriented such that lower values indicate better performance and evaluated on an additional test data set of size $n_{\text{test}}=1000$ observations that are not used in the fitting process or for tuning. The energy score is computed using the scoringRules^33^ package. We include univariate distribution-specific evaluation criteria as well, although we remark that these criteria do not take the dependence between the responses into account. For binary responses, we use the Brier score and the area under the curve. For the remaining discrete and mixed responses we compute the univariate mean squared error of prediction comparing the true $Y_{j}$ with its prediction ${\hat{Y}}_{j}$ , j=1,2 . The bivariate observations are generated using the VineCopula^34^ package. All performance measures are averages over the observations in the test set and averages over 200 independent data set replications. Lastly, we report the selection rates of the informative and non-informative variables for each distributional parameter. The selection rates are defined as the percentage of simulation replications in which the informative/non-informative variables have been selected, averaged by the number of informative/non-informative variables in each distribution parameter, respectively.

### 3.2. Details of sparsity and dimensions

To challenge the boosting algorithm, we consider different amounts of sparsity and covariates that are informative in more than one distribution parameter. For the bivariate binary response scenario (Section SB1 in Supplemental material B) $p_{1}=100,p_{2}=100,\text{ and }p_{3}=1000$ candidate covariates are considered. Only six covariates have a linear effect on the bivariate distribution, whereas the rest are noise variables. This leads to $50\%\;(p_{1}),5\%\;(p_{2})\text{ and }0.5\%\;(p_{3})$ of the candidate covariates being informative, respectively, thereby reflecting low, medium and high levels of sparsity. The scenario with bivariate counts (Section SB2 in Supplemental material B) is comprised of linear and non-linear data-generating processes (DGPs) with $p_{1}=10$ independent variables. In these configurations 60%(linear DGP) and 50%(non-linear DGP) of the covariates were informative. The mixed binary & continuous scenario (Section SB3 in Supplemental material B) consists of linear and a non-linear DGPs with $p_{1}=10$ . In those simulations, 50%(linear DGP) and 30%(non-linear DGP) of the covariates were informative. With $n_{\text{train}}=1000$ throughout, all but the SB1/ $p_{3}$ case, where p=n , are low-dimensional settings with p<n .

### 3.3. Overall summary of simulation results

In general, the performance of the proposed boosted copula models is satisfactory. They effectively detect and recover all effects across different parameters of the bivariate distribution. Notably, the copula dependence parameter shows a stronger shrinkage of informative effects compared to other parameters. As the number of considered covariates increases, the degree of shrinkage also rises. This behaviour may be attributed to the greedy nature of the algorithm, since a reduction of the loss from including a covariate with a small coefficient in the dependence parameter might not be large enough compared to updating a coefficient in any other parameter corresponding to the margins. Consequently, this can lead to sparser dependence parameters with relatively small effects being falsely disregarded. The choice of ${\text{m}}_{\text{stop}}$ in the distribution parameter of the copula remains an under-explored area, deserving attention in future research to address this issue.

Overall, the copula approach is competitive in terms of selection rates of covariates in the marginal parameters and satisfactory in identifying the most relevant effects in the dependence parameter. Based on scores evaluating the predictive behaviour of the joint distribution, the added value compared to using boosting with independent univariate models becomes obvious even for moderate associations between the response components.

## 4. Biomedical applications

In this section, we illustrate the versatility of our proposed boosted distributional copula regression approach by analysing three different biomedical research questions. In Section 4.1, we model the joint distribution of two binary responses which correspond to the presence of heart disease (yes/no) as well as the presence of high cholesterol (yes/no) using data from the large-scale biomedical database UK Biobank genetic cohort study^35^ under application number 81202. This corresponds to a high-dimensional setting in the covariate space. In Section 4.2, we are concerned with the joint distribution of a bivariate count vector comprised of the number of doctor consultations and the number of prescribed medications from Australian healthcare recipients using data from the R package bivpois.^36^ We demonstrate how to conduct model-building when the choice of marginal distributions, as well as copula functions, is not clear. Lastly, in Section 4.3, we investigate the distribution of two mixed responses relevant for analysing infant undernutrition in India emanating using data from the Demographic and Health Survey (DHS; https://dhsprogram.com, accessed on 13 December 2023).^37^ In what follows, the step-length of the boosting algorithm is set to ${\text{s}}_{\text{step}}=0.1$ and the number of fitting iterations ${\text{m}}_{\text{stop}}$ is optimized via the predictive or out-of-bag risk as outlined in Step 4 of Algorithm 1 in Supplemental material A. We resort to $L_{2}$ -stabilisation in order to achieve similar effective step-lengths across the different parameters of the bivariate distributions.

### 4.1. Chronic ischaemic heart disease and high cholesterol

We analyse a subsample consisting of n=30,000 individuals and p=1867 pre-filtered genetic variants (covariates). This sample has been previously analysed in Strömer et al.^17^ using a bivariate Bernoulli distribution. The responses are the presence of chronic ischaemic heart disease (CIHD), and high cholesterol (cholesterol), both encoded as binary variables. The prevalence of the two factors in our sample is 7.2% and 32.3%, respectively.

#### 4.1.1. Model specification

We build the joint distribution using a Gaussian copula with logit margins. We split the sample into two partitions dedicated for fitting ( $n_{\text{train}}=20,000$ ) and tuning of ${\text{m}}_{\text{stop}}$ ( $n_{\text{mstop}}=10,000$ ). The additive predictors of the bivariate distribution are

$$\eta_{i1}^{\left(∙\right)}=\beta_{01}^{\left(∙\right)}+\sum_{r=1}^{1,867}\beta_{r1}^{\left(∙\right)}x_{ir},\;\text{ with }∙=\{1,2,c\}$$

#### 4.1.2. Results

**Figure 2.**
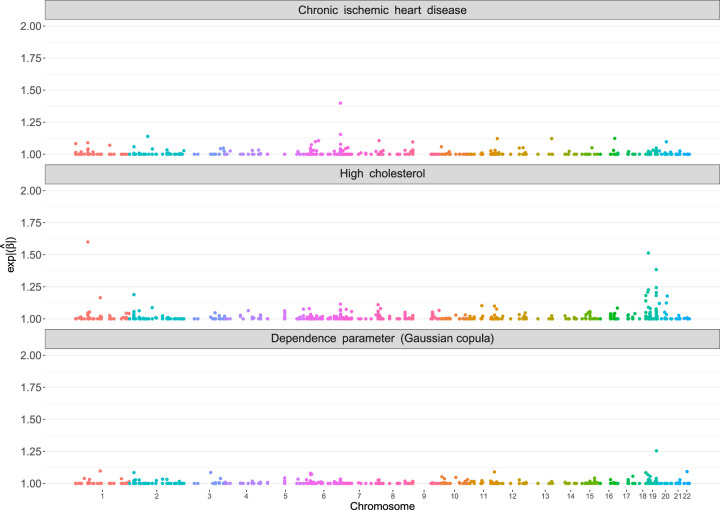
Application in Section 4.1. Manhattan-type plots of the estimated coefficients (expressed in exponential absolute values of the estimated values) of the boosted bivariate binary model using a Gaussian copula. The x -axis represents the genomic location of the variants and the y -axis shows $\exp\;(|{\hat{\beta }}_{j}|)$ , j=1,…,p .

The estimated coefficients, expressed as the exponential absolute values in each margin and the dependence parameter, are shown in a Manhattan-type plot^38^ in Figure 2. The scale of the y -axis of the Manhattan plot has been modified to reflect the importance of the different genetic variants via the exponential absolute value of the estimates coefficients (for the margins, similar to an odds ratio from logistic regression). Using the estimated dependence parameters ${\hat{\vartheta }}_{i}^{\left(c\right)}$ , for i=1,…,n , we compute the corresponding Kendall’s τ , which range from $\hat{\tau }\in [-0.567;\;0.289]$ . This result indicates that there is a moderate negative dependence between the probabilities of chronic heart disease and high cholesterol. This finding most likely reflects the common use of statins in the population of patients already diagnosed with chronic heart disease.^39^ Our proposed boosting method selects several variants in the respective parameters of the bivariate distribution. For instance, out of a potential 1867 possible candidates, 140 variants are selected in the first margin ( $\vartheta_{1}^{\left(1\right)}$ ), 322 variants in the second margin ( $\vartheta_{1}^{\left(2\right)}$ ) and 181 in the dependence parameter $\vartheta^{\left(c\right)}$ with some overlap in the selected variants between the parameters (90 variants selected for two out of three parameters). A total of 19 variants are shared between the dependence parameter and $\vartheta_{1}^{\left(1\right)}$ , whereas $\vartheta_{1}^{\left(2\right)}$ and $\vartheta^{\left(c\right)}$ have 48 variants in common. Moreover, 23 variants are shared among the margins. The findings of our copula model agree with previous studies on the location of cholesterol-associated genes, see, for example, Richardson et al.,^40^ where the highest estimated coefficient values are present.

### 4.2. Doctor consultations and prescribed medications in Australia

We study the joint distribution of a bivariate count response comprised of the number of doctor consultations ( doctorco ) and the number of prescribed medications ( prescrib ) of healthcare recipients from Australia. The sample consists of n=5190 observations and we use 65% of them to fit the model ( $n_{\text{train}}=3114$ ), and 25% for optimising ${\text{m}}_{\text{stop}}$ ( $n_{\text{mstop}}=1298$ ). An additional test partition of $n_{\text{test}}=778$ observations is used to determine the best-fitting marginal distributions and copula function. The dataset comprises two continuous covariates. These are age (age in years divided by 100) and income (annual income in Australian dollars divided by 1000). In addition, the binary covariate gender (1 female, 0 male) is reported.

#### 4.2.1. Marginal distributions

The best-fitting marginal distributions have been determined via the out-of-sample negative log-likelihood on the test partition of the data, see Table SC1 in Supplemental material C for more details. As shown in Figure 1(b), each of the marginal responses exhibits a large amount of zeros and their respective variances differ from the mean ( $\bar{\text{doctorco}}=0.302$ ; Var(doctorco)=0.637 , and $\bar{\text{prescrib}}=0.863$ ; Var(prescrib)=2.003 ). While these descriptive statistics do not account for the covariates, we also find that with regressors the Poisson distribution is not suited to model the conditional distribution of the two responses. The best-fitting marginal distributions in terms of the out-of-sample negative log-likelihood are the zero-altered logarithmic distribution $(\vartheta_{1}^{\left(1\right)},\vartheta_{2}^{\left(1\right)}{)}^{⊤}$ for doctorco, where the probability of observing a zero is modelled by the parameter $\vartheta_{2}^{\left(1\right)}$ . The zero-inflated negative binomial distribution $(\vartheta_{1}^{\left(2\right)},\vartheta_{2}^{\left(2\right)},\vartheta_{3}^{\left(2\right)}{)}^{⊤}$ is the most suitable for prescrib. With this, the probability of observing a zero is explicitly modelled via the parameter $\vartheta_{3}^{\left(2\right)}$ .

#### 4.2.2. Copula selection

The copula was selected by means of the out-of-sample negative log-likelihood (using the same test data as for the margins) out of six possible candidates: Gaussian, Frank, Clayton, Gumbel, Farlie–Gumbel–Morgenstern and Ali–Mikhail–Haq copulas, with the Clayton copula giving the best out-of-sample negative log-likelihood. This indicates that the data support the presence of lower tail dependence, that is, strong dependence of very low values in both marginal responses. In addition, the Clayton copula performs better than independent margins as well as the bivariate Poisson distribution, see Table SC2 in Supplemental material C for more details.

#### 4.2.3. Predictor specification

As a result of the selection of marginal distributions, there are six parameters in the bivariate distribution ( $K_{1}=2,\;K_{2}=3,\;K_{c}=1$ ) and all additive predictors in the distribution share the following configuration:

ηik(∙)=β0k(∙)+β1k(∙)genderi+s1k(∙)(incomei):genderi+=s2k(∙)(agei):genderi+s3k(∙)(incomei,agei),=∀k=1,…,K∙,∙∈{1,2,c}

where the term s(⋅):gender denotes a varying coefficient term, where age or income are effect modifiers of the effect of gender , respectively. The base-learner $s_{3}^{\left(∙\right)}(\text{income},\;\text{age})$ indicates the interaction term of the respective covariates and is specified as a two-dimensional P-spline. Therefore, this configuration takes into account the main effect of all covariates in the data as well as possible interactions between them.

#### 4.2.4. Results

Table SC3 in Supplemental material C shows the base-learners selected in each parameter of the joint bivariate distribution. The fitted values of the dependence expressed as Kendall’s τ range within $\hat{\tau }\in [0.341;\;0.539]$ , indicating a moderate estimated dependence between the margins in the sample, conditional on all selected covariate effects. Only the main effect of age and gender were selected on the copula dependence parameter.

**Figure 3.**
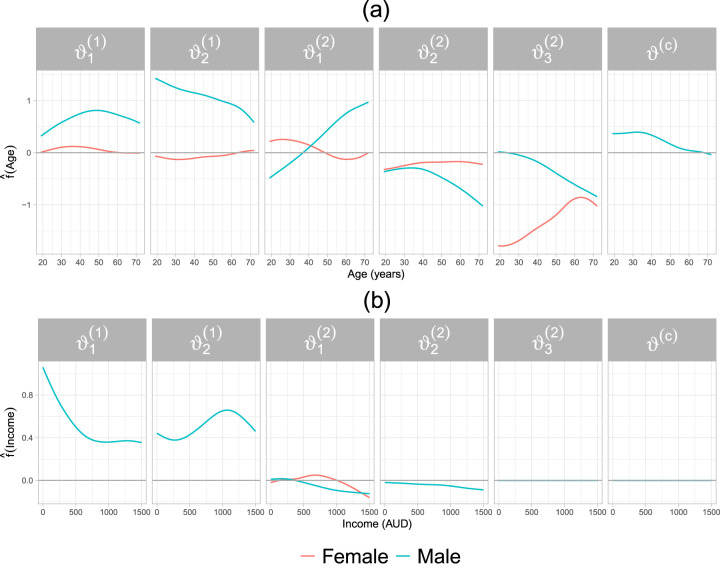
Application in Section 4.2. Estimated partial effects of age (a) and income (b) on the additive predictors $\eta_{k}^{\left(∙\right)}$ of the parameters of the marginal distributions as well as the dependence parameter of a Clayton copula.

The results of non-linear effect estimates and selected effect modifiers are depicted in Figure 3. The covariate age has a non-zero effect in all parameters of the bivariate distribution (see panel (a) of Figure 3) and it interacts with gender only on the marginal distributions. In particular, the effect of age on $\vartheta_{1}^{\left(1\right)}$ is increasing between 20 and 50 years, and then becomes decreasing for older male individuals. For female individuals, the effect follows a similarly shaped pattern, albeit the positive effect lasts until the mid-30s and the range of the effect is close to zero. Increasing age leads to smaller values of the parameter $\vartheta_{2}^{\left(1\right)}$ for male individuals, whereas for females the effect leads to an increase in $\eta_{2}^{\left(1\right)}$ but its range is once again close to zero. The two aforementioned parameters jointly determine the expectation and variance of doctorco, whereas the parameter $\vartheta_{2}^{\left(1\right)}$ explicitly models the probability of observing a zero. Hence, for older individuals, it becomes less likely to have zero doctor consultations.

**Figure 4.**
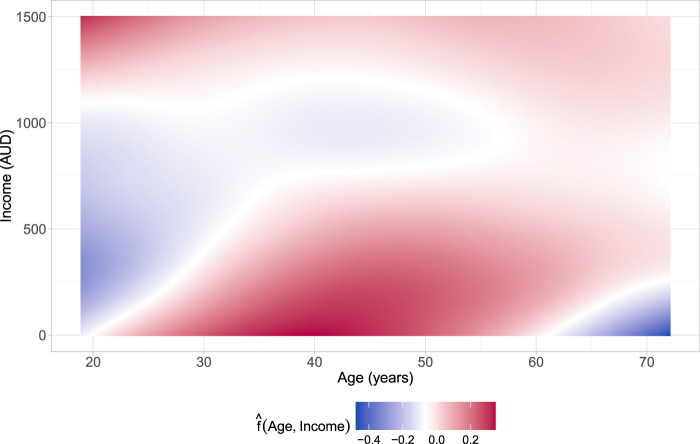
Application in Section 4.2. Estimated partial interaction effect of age and income on the additive predictor $\eta_{1}^{\left(2\right)}$ of the parameter $\vartheta_{1}^{\left(2\right)}$ (number of prescribed medications; prescrib).

For male individuals, increasing age leads to an increase in the predictor of $\vartheta_{1}^{\left(2\right)}$ , which partially determines the expected number of prescribed medications. Intuitively, the predictor of $\vartheta_{3}^{\left(2\right)}$ decreases almost linearly with the male individual’s age, which directly translates to the logit of a decreased probability of observing a zero in the second margin. In other words, older male individuals are more likely to have a number of prescribed medications that are larger than zero. Conversely, the effect of age of female individuals on the predictor of $\vartheta_{3}^{\left(2\right)}$ shows an upward trend, which indicates an increasing likelihood of having zero prescribed medications. A downward-sloping effect of age is estimated for the parameter $\vartheta_{2}^{\left(2\right)}$ for both female and male individuals. Additionally, the dependence between the margins decreases in older individuals as seen in the panel corresponding to $\vartheta^{\left(c\right)}$ . Note that the interaction between age and gender is not selected in the dependence parameter.

The covariate income is selected in four parameters of the bivariate distribution, see Figure 3(b). The individual’s income has a non-zero effect on the parameters of doctorco distribution and shows no interaction with gender. Conversely, income exhibits a much smaller, albeit downward-sloping, effect on the parameters $\vartheta_{1}^{\left(2\right)}$ and $\vartheta_{2}^{\left(2\right)}$ of the distribution of prescrib. The interaction of income and gender is selected only on the parameter $\vartheta_{1}^{\left(2\right)}$ . The covariate income was neither selected on the parameter $\vartheta_{3}^{\left(2\right)}$ nor on the dependence parameter. This result indicates that income does not play a role in the association between prescrib and doctorco. The interaction between age and income is only selected for the parameter $\vartheta_{1}^{\left(2\right)}$ . The estimated two-dimensional P-spline depicted in Figure 4 shows that there is an interplay between an individual’s age and income on the expected number of prescribed medications (prescrib). For younger individuals with low to moderate income, the interaction reduces the value of the additive predictor of $\vartheta_{1}^{\left(2\right)}$ . A similar pattern can be observed for individuals in a higher age bracket ( ≥70 years) with low income.

The covariate gender was selected in all parameters except for $\vartheta_{1}^{\left(1\right)}$ , see Table 1, middle block. The estimates of gender in the first margin indicate that the expected value of both responses is higher for female healthcare recipients, *ceteris paribus*. This is due to $\vartheta_{2}^{\left(1\right)}$ directly modelling the probability of observing no doctor visits. The estimated effect of gender in $\vartheta_{3}^{\left(2\right)}$ also suggests that the probability of having zero prescribed medications is lower for female recipients compared to male individuals. Lastly, the dependence between the margins is lower for female individuals, relative to their male counterparts.

**Table 1.** Estimated linear effects for applications in Sections 4.1) (first block), 4.2 (second block) and 4.3 (third block) across distribution parameters.

		Margin 1		Margin 2			Copula
Application	Covariate	$\vartheta_{1}^{\left(1\right)}$	$\vartheta_{2}^{\left(1\right)}$	$\vartheta_{1}^{\left(2\right)}$	$\vartheta_{2}^{\left(2\right)}$	$\vartheta_{3}^{\left(2\right)}$	$\vartheta^{\left(c\right)}$
Bivariate binary		Bernoulli (logit)		Bernoulli (logit)			Gaussian
	Intercept	−1.198	–	−0.317	–	–	−0.442
Bivariate count		ZALG		ZINBI			Clayton
	Intercept	−1.193	−0.050	−0.255	0.234	0.022	−0.452
	gender (female)	0	−0.218	0.189	−0.447	−1.171	−0.379
Bivariate mixed		Bernoulli (probit)		Gaussian			Clayton $270^{\circ }$
	Intercept	−0.230	–	0.003	0.008	–	0
	cgender (female)	−0.031	–	0	0.002	–	0

The symbol “–” indicates that the distribution does not feature the respective parameter, whereas 0 indicates that the algorithm did not select the respective covariate.

**Figure 5.**
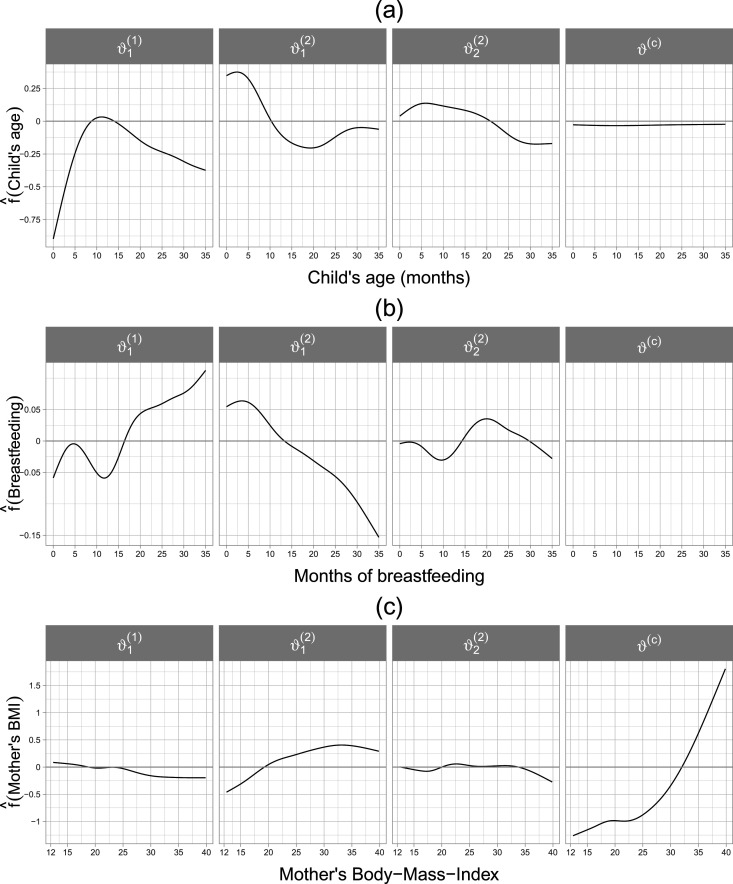
Application in Section 4.3. Estimated partial effects of the child’s age (cage, a), months of breastfeeding (breastfeeding, b) and the mother’s body-mass-index (mbmi, c) on the additive predictors $\eta_{k}^{\left(∙\right)}$ of the parameters of the margins as well as the dependence parameter of a Clayton copula rotated by $270^{\circ }$ .

**Figure 6.**
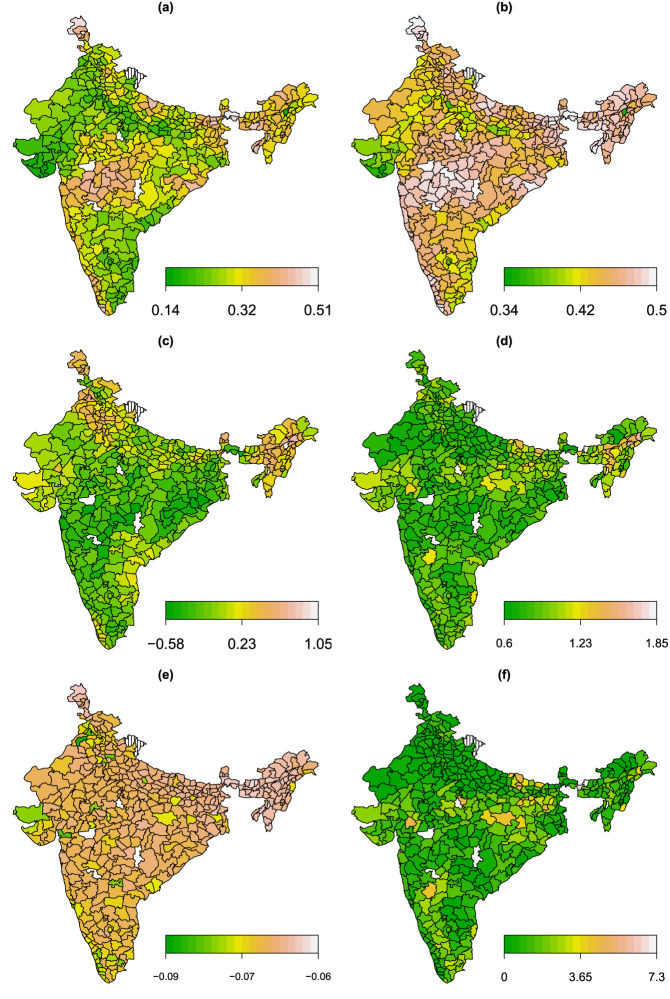
Application in Section 4.3. Shown are (a) expected value, (b) standard deviation of fever; (c) estimated expected value, (d) standard deviation of wasting; (e) estimated Kendall’s τ , and (f) joint probabilities in % of having fever and moderate undernutrition according to the Clayton copula rotated by $270^{\circ }$ .

### 4.3. Determinants of infant undernutrition in India

We analyse a sample of n=24,286 observations to study jointly two determinants of child undernutrition in India. The binary response fever indicates whether a child has had fever up to two weeks prior to the survey interview, whereas wasting denotes low weight-for-height, indicating an acute recent weight loss. According to UNICEF, this is the most immediate, visible and life-threatening form of undernutrition.^41^ The individuals in the sample are spread across 438 administrative units (districts) with some imbalance in the number of observations per district. We resort to a slightly different sub-sampling scheme compared to the previous applications in order to obtain $n_{\text{train}}$ , and $n_{\text{mstop}}$ . We include all observations corresponding to districts with a sample size below or equal to 40 in $n_{\text{train}}$ . For all other districts with more than 40 observations, we sample without replacement and obtain a fraction of around 75% of the total observations used for training ( $n_{\text{train}}=18,214$ ) and 25% for optimizing mstop ( $n_{\text{mstop}}=6072$ ). Table SC4 (Supplemental material C) summarizes the responses and available covariates.

#### 4.3.1. Model specification

We follow Klein et al.^15^ and set the link function for the model of fever to probit, whereas for wasting we resort to a heteroscedastic Gaussian distribution. The dependence between the margins is modelled using a Clayton copula rotated by $270^{\circ }$ . This allows us to model dependence between very high values of fever and very low values of wasting. It seems reasonable to expect such a dependence structure to be supported by the data, since it is likely that the probability of children experiencing fever is prone to be dependent on low weight-for-height values (wasting, i.e. undernourished infants). Consequently, the bivariate distribution has K=4 distribution parameters. In Klein et al.,^15^ the additive predictor of the margins was fixed and an information-criterion-based model selection procedure was conducted using different configurations of the predictor of $\vartheta^{\left(c\right)}$ . Here we allow our proposed approach to select the variables in all predictors of the bivariate distribution in a data-driven manner, without further input from the analyst. That is,

ηik(∙)=β0k(∙)+β1k(∙)cgenderi+s1k(∙)(cagei)+s2k(∙)(mbmii)+=s3k(∙)(breastfeedingi)+s4k(∙)(distHi)

where k=1,…,K,∙∈{1,2,c} and $s_{4k}^{\left(∙\right)}({\text{distH}}_{i})$ is set as a Markov random field base-learner to model the discrete spatial information of the districts in the data. The covariates cage, mbmi and breastfeeding are incorporated using P-spline base-learners with 20 knots and second-order difference penalties, whereas a linear base-learner is used for cgender.

#### 4.3.2. Results

The estimated dependence between the margins in terms of Kendall’s τ ranges within $\hat{\tau }\in [-0.561;\;-0.052]$ , suggesting a negative dependence between wasting and fever. This is a reasonable finding since a lower wasting score implies a more severe form of undernutrition, whereas the risk of fever is expected to be positively associated with poor health status. The estimated non-linear effects of the covariates cage, breastfeeding and mbmi are visualized in Figure 5. It can be seen that children within 0 and ≈12 months of age have an increasing likelihood of fever. The estimated effect of cage is downward-sloping in the first 20 months on the expectation of wasting, whereas on the standard deviation, a similar pattern is observed albeit with a much smaller slope, see Figure 5(a). In terms of the dependence structure, the child’s age appears to have a negligible effect. The estimated effect of breastfeeding on fever depicted in Figure 5(b) shows an upward slope and on $\vartheta_{1}^{\left(1\right)}$ a downward slope. The presence of breastfeeding at a later age of the child could reflect a lack of other sources of nourishment apart from the mother, serving as a proxy for household’s poverty, thus driving the probability of fever upwards and the expected value of wasting downwards. The variable breastfeeding is not selected in the dependence parameter. Compared to cage and breastfeeding, the mother’s body-mass-index (mbmi) shows a small to moderate (see $\vartheta_{1}^{\left(2\right)}$ ) association with the margins, see Figure 5(c). The effect of mbmi is slightly increasing in the expectation of wasting and remains stable at around mbmi≈25 . However, the effect of mbmi leads to a sharp increase in the dependence between the margins after it reaches values of approximately 25. The covariate cgender was not selected in $\vartheta_{1}^{\left(2\right)}$ as well as $\vartheta^{\left(c\right)}$ and it shows a very small value in $\vartheta_{2}^{\left(2\right)}$ , compare Table 1, third block. Finally, Figure 6 presents various estimated quantities (expectation, standard deviation and Kendall’s τ , joint probabilities) according to the spatial structure of the data. The spatial component modelling the districts (distH) is selected in all parameters. In Figure 6(a) it can be observed that the districts located in the centre of India exhibit a higher probability of fever, however, the standard deviation of fever is rather high across the country (see Figure 6(b)). The expectation of wasting remains mostly low throughout all districts, with some exceptions located in the north and north-eastern districts of India, see Figure 6(c). Compared to fever, the standard deviation of wasting is rather low in most districts, see Figure 6(d). Figure 6(e) and (f) visualizes the per district average of the estimated dependence between the margins in terms of Kendall’s τ and the estimated joint probabilities (in %) of having fever and moderate undernutrition, that is, $P(Y_{1}=1,Y_{2}<-2)$ . It can be seen that the magnitude of the dependence is larger in some districts located in the north-western are, as well as the south-eastern coast of India. The joint probabilities of fever and moderate undernutrition indicate that children located in mid-eastern districts are more prone to suffer from undernutrition.

## 5. Discussion

We have extended the boosted distributional copula regression approach^16^ to accommodate arbitrary response types on different domains. We conducted a wide range of simulation studies to investigate the predictive performance, as well as the estimation capabilities of our proposed method. Overall, we found that our approach outperforms univariate boosting models when it comes to probabilistic forecasting for the joint bivariate distribution.

We were able to demonstrate that our proposed copula approach allows us to capture the nuances of each marginal response, such as zero-inflation, over-dispersion, or heteroscedasticity, while also modelling the dependence between the margins using only one statistical model. Additionally, our methodology and software implementation allow us to conduct data-driven variable selection without further input from the analyst as well as transparent and reproducible research.

We have illustrated the application of our approach on three diverse biomedical datasets. In the first application, we identified relevant genetic variants associated with the dependence of high cholesterol and ischaemic heart disease. Although not conducted here due to computation time constraints, other copula functions than the Gaussian copula could be tested in order to investigate whether the data support lower or upper tail dependence. In our second healthcare-related application, we found that data on the number of doctor consultations and number of prescribed medications support lower tail dependence, that is, dependence between extremely low values of the margins. Finally, in the third application, we studied the joint distribution of two determinants of infant undernutrition that emanate from different domains. One determinant is expressed as a binary indicator whereas the other is a continuous marker.

While our approach is very useful for conducting explanatory analyses and for predictive modelling, the main limitation of resorting to statistical boosting for model fitting is the lack of confidence intervals for the estimated effects. While in principle access to these is possible using bootstrap methods, doing so is a cumbersome and time-consuming task. Another limitation was observed in our simulation studies in Section 3. The boosted models have a tendency to select false positives throughout the fitting process and the different distribution parameters. Although the estimated effect of these false positives is in most cases small or negligible, a formal correction of these incorrectly estimated effects would be appealing. An adaptation of the de-selection procedure implemented by Strömer et al.^42^ would lead to more sparse models and stable selection of informative covariates.

Another future field of application where data-driven variable selection can have a big impact is in observational studies where endogenous variables are present, see, for example, Briseño Sanchez et al.^43^ and Wyszynski et al.^44^ Statistical boosting could provide valuable insights in these scenarios, since the effect of endogenous variables is identifiable as long as so-called instruments are available, which boosting could help identify and to validate the analyst’s beliefs. Lastly, we are also exploring an extension of our boosting methodology to fit distributional copula regression models for bivariate time-to-event data, which would greatly extend the applicability of our software implementation in biomedical research: In clinical applications, the interest may lie in overall survival expressed as a time of a landmark event (e.g. tumour progression), time of death, or another event time associated with a patient’s condition or chronic disease. The issue in most time-to-event applications is the presence of censoring, posing a challenge for estimation. It could be argued that copula regression models for standard continuous outcomes would be applicable for time-to-event data, albeit under the absence of censoring – which is unrealistic in most practical scenarios. While some statistical packages such as GJRM^45^ and joint.Cox^46^ offer a wide range of functionality and flexibility for bivariate time-to-event data, but they lack the ability to conduct data-driven variable selection. Therefore, extending our proposed methodology also to multivariate censored time-to-event outcomes could help to fill this gap in medical research.

## Supplemental Material

sj-pdf-1-smm-10.1177_09622802241313294 - Supplemental material for Boosting distributional copula regression for bivariate binary, discrete and mixed responses Supplemental material, sj-pdf-1-smm-10.1177_09622802241313294 for Boosting distributional copula regression for bivariate binary, discrete and mixed responses by Guillermo Briseño Sanchez, Nadja Klein, Hannah Klinkhammer and Andreas Mayr in Statistical Methods in Medical Research
