# Supplementary material for: Boosting distributional copula regression for bivariate binary, discrete and mixed responses
Source: Stat Methods Med Res. 2025 Mar 21;34(5):887–902. doi: 10.1177/09622802241313294 (PMC12177205; doi:10.1177/09622802241313294)
Supplement: sj-pdf-1-smm-10.1177_09622802241313294 - Supplemental material for Boosting distributional copula regression for bivariate binary, discrete and mixed responses [file sj-pdf-1-smm-10.1177_09622802241313294.pdf]

Supplementary Material

for

“Boosting distributional copula regression for bivariate binary, discrete and mixed responses”

Contents

Part A: Details on implemented boosting algorithm, copula functions, marginal distributions and negative gradients of the implemented loss functions.

Part B: Details and results of the simulation study.

Part C: Additional results and supporting information of the biomedical applications.

Part A

This supplement contains the detailed description of our boosting algorithm for distributional copula regression with faster tuning of the number of fitting iterations as well as the definition of implemented copulas and marginal distributions. In addition, it contains the tables that define the implemented copula functions and marginal distributions. Lastly, it shows the definition of the negative gradients of the implemented loss functions.

Algorithm 1 Non-cyclic boosting for distributional copula regression with faster tuning of fitting iterations $\mathtt{m_{stop}}$ by means of out-of-bag (*oobag*) risk.

Require:

Define the base-learners $b_r^{(\bullet)}(x_r)$ for $r = 1, \ldots, P_{vk}$, $\bullet = 1, 2, c$.

Set the step-length $\mathtt{s_{step}} \ll 1$ as well as the (non-optimal) number of fitting iterations $\mathtt{m_{stop}}$.

Set weights indicating the training and $\mathtt{m_{stop}}$-tuning partitions of the sample n_{train}, $n_{\mathtt{mstop}}$.

Set type of stabilisation to be applied to the negative gradient vector (L_2, median absolute deviation or none).

(1) Initialise all predictors $\hat{\eta}_k^{(\bullet)}$ corresponding to $\vartheta_k^{(\bullet)} \in \boldsymbol{\vartheta}$ with offset values $\hat{\eta}_{k,[0]}^{(\bullet)}$.

for $m = 1, \ldots, \mathtt{m_{stop}}$ **do**

for $k = 1, \ldots, K$ in $\vartheta_k^{(\bullet)} \in \boldsymbol{\vartheta}$ **do**

(a) Evaluate the parameter-specific negative gradient vector $-\boldsymbol{g}_{k,[m]}^{(\bullet)}$

$$-\boldsymbol{g}_{k,[m]}^{(\bullet)} = \left(-\boldsymbol{g}_{k,[m]}^{(\bullet)}(\boldsymbol{x}_i)\right)_{i=1,\ldots,n_{\text{train}}} = -\left(\left. \frac{\partial \omega\left(\boldsymbol{y}_i, \hat{\boldsymbol{\eta}}_i\right)}{\partial \eta_k^{(\bullet)}} \right|_{\hat{\boldsymbol{\eta}} = \hat{\boldsymbol{\eta}}_{[m-1]}(\boldsymbol{x}_i)} \right)_{i=1,\ldots,n_{\text{train}}}.$$

(b) Fit $-\boldsymbol{g}_{k,[m]}^{(\bullet)}$ to each parameter-specific base-learner $b_{k,j}^{(\bullet)}(x_j)$.

(c) Select the best-fitting base-learner $\hat{b}_{k,j^\star}^{(\bullet)}$ via residual sum of squares criterion.

$$j^\star = \underset{j \in 1,\ldots P_k^{(\bullet)}}{\arg\min} \sum_{i=1}^{n_{\text{train}}} \left(-g_{k,[m]}^{(\bullet)}(\mathbf{x}_i) - \hat{b}_{k,j}^{(\bullet)}(x_i)\right)^2.$$

(d) Compute loss reduction of a weak update using $\hat{b}_{k,j^\star}^{(\bullet)}$.

$$\Delta\omega_{\vartheta_k^{(\bullet)}} = \sum_{i=1}^{n_{\text{train}}} \omega\left(\boldsymbol{y}_i; \hat{\eta}_k + \mathtt{s_{step}} \hat{b}_{k,j^\star}^{(\bullet)}(x_{ij^\star})\right).$$

end for

(2) Update the parameter with highest loss reduction $\vartheta_k^{(\bullet)^\star} = \arg\min_{\vartheta_k^{(\bullet)} \in \boldsymbol{\vartheta}} \left(\Delta\omega_{\vartheta_k^{(\bullet)}}\right)$:

$$\hat{\eta}_{k,[m]}^{(\bullet)*}(\boldsymbol{x}_i) = \hat{\eta}_{k,[m-1]}^{(\bullet)*}(\boldsymbol{x}_i) + \mathtt{s_{step}} \cdot \hat{b}_{k,j^\star}^{(\bullet)}(x_{ij^\star}).$$

(3) For the remaining parameters $\vartheta_k^{(\bullet)} \neq \vartheta_k^{(\bullet)^\star}$, set $\hat{\eta}_{k,[m]}^{(\bullet)}(\boldsymbol{x}_i) = \hat{\eta}_{k,[m-1]}^{(\bullet)}(\boldsymbol{x}_i)$.

(4) Compute the out-of-bag risk at iteration $[m]$:

$$\text{risk}_{\text{oobag},[m]} = \sum_{i=1}^{n_{\mathtt{mstop}}} \hat{\omega}\left(\boldsymbol{y}_i; \hat{\boldsymbol{\eta}}_{\boldsymbol{i}}|_{\hat{\eta} = \hat{\eta}_{[m]}(\boldsymbol{x}_i)}\right).$$

end for

(5) Determine $\mathtt{m_{stop}^{opt}}$ by means of the out-of-bag risk:

$$\mathtt{m_{stop}^{opt}} = \underset{m \in 1,\ldots,\mathtt{m_{stop}}}{\arg\min} \text{risk}_{\text{oobag},[m]}.$$

Table A1: Details of implemented copulas. The functions $\Phi_1^{-1}(\cdot)$ and $\Phi_2(\cdot)$ denote the quantile function and CDF of the univariate and bivariate standard normal distributions, respectively. Rotated copulas by 90, 180 and 270 degrees are respectively defined as: $C_{90} = F_2 - C(1 - F_1, F_2; \vartheta^{(c)})$, $C_{180} = F_1 + F_2 - 1 + C(1 - F_1, 1 - F_2; \vartheta^{(c)})$ and $C_{270} = F_1 - C(F_1, 1 - F_2; \vartheta^{(c)})$. The term $D_1(\vartheta^{(c)}) = \int_0^{\vartheta^{(c)}} \frac{t}{\exp(t)-1} dt$ is the Debye function and Φ_2 denotes the CDF of the bivariate Gaussian distribution with correlation coefficient $\vartheta^{(c)}$. Finally, AMH stands for Ali-Mikhail-Haq and FGM stands for Farlie-Gumbel-Morgenstern.

Copula	$C(F_1, F_2; \vartheta^{(c)})$	Range of $\vartheta^{(c)}$	Link	Kendall's τ
Gauss	$\Phi_2(\Phi_1^{-1}(F_1), \Phi_1^{-1}(F_2); \vartheta^{(c)})$	$\vartheta^{(c)} \in [-1, 1]$	$\tanh^{-1}(\vartheta^{(c)})$	$\frac{2}{\pi} \arcsin(\vartheta^{(c)})$
Clayton	$(F_1^{-\vartheta^{(c)}} + F_2^{-\vartheta^{(c)}} - 1)^{-1/\vartheta^{(c)}}$	$\vartheta^{(c)} \in (0, \infty)$	$\log(\vartheta^{(c)})$	$\frac{\vartheta^{(c)}}{\vartheta^{(c)}+2}$
Gumbel	$\exp\left[-\left\{(-\log(F_1))^{\vartheta^{(c)}} + (-\log(F_2))^{\vartheta^{(c)}}\right\}^{\frac{1}{\vartheta^{(c)}}}\right]$	$\vartheta^{(c)} \in [1, \infty)$	$\log(\vartheta^{(c)} - 1)$	$1 - \frac{1}{\vartheta^{(c)}}$
Frank	$-{\vartheta^{(c)}}^{-1} \log\Big(1 + (\exp(-\vartheta^{(c)} F_1) - 1) \cdot$	$\vartheta^{(c)} \in \mathbb{R} \setminus \{0\}$	$\vartheta^{(c)}$	$1 - \frac{4}{\vartheta^{(c)}}[1 - D_1(\vartheta^{(c)})]$
	$(\exp(-\vartheta^{(c)} F_2) - 1)/(\exp(-\vartheta^{(c)}) - 1)\Big)$			
AMH	$F_1 F_2/(1 - \vartheta^{(c)}(1 - F_1)(1 - F_2))$	$\vartheta^{(c)} \in [-1, 1]$	$\tanh^{-1}(\vartheta^{(c)})$	$1 - \frac{2}{3}{\vartheta^{(c)}}^2(\vartheta^{(c)} + (1 - \vartheta^{(c)})^2 \log(1 - \vartheta^{(c)}))$
FGM	$F_1 F_2/(1 + \vartheta^{(c)}(1 - F_1)(1 - F_2))$	$\vartheta^{(c)} \in [-1, 1]$	$\tanh^{-1}(\vartheta^{(c)})$	$\frac{2}{9}\vartheta^{(c)}$
Joe	$1 - ((1 - F_1)^{\vartheta^{(c)}} + (1 - F_2)^{\vartheta^{(c)}} - (1 - F_1)^{\vartheta^{(c)}}(1 - F_2)^{\vartheta^{(c)}})^{(1/\vartheta^{(c)})}$	$\vartheta^{(c)} \in [1, \infty)$	$\log(\vartheta^{(c)} - 1)$	$1 + \frac{4}{{\vartheta^{(c)}}^2} \int_0^1 x \log(x)(1 - x)^{2(1-\vartheta^{(c)})/\vartheta^{(c)}} dx$

Table A2: Details of newly implemented univariate marginal distributions for binary, continuous and discrete responses to be used together with copulas in the `gamboostLSS` package. For the Zero-Altered Logarithmic distribution, the term $\alpha = -[\log(1-\vartheta_1)]^{-1}$. For the Zero-Altered Negative Binomial distribution, the term $c = \frac{1-\vartheta_3}{\left(1-(1+\vartheta_1\vartheta_2)^{-1/\vartheta_2}\right)}$. All distributions use the parameterisation from Rigby et al. (2019).

Distribution	$E(Y)$	$Var(Y)$	Parameters & range	Links	Response range
Bernoulli	ϑ_1	$\vartheta_1(1-\vartheta_1)$	$\vartheta_1 \in [0,1]$	logit<br>probit<br>cloglog	$y \in \{0,1\}$
Gaussian	ϑ_1	ϑ_2^2	$\vartheta_1 \in \mathbb{R}, \vartheta_2 > 0$	Identity, log	$y \in \mathbb{R}$
Poisson	ϑ_1	ϑ_1	$\vartheta_1 > 0$	log	$y \in \mathbb{N}_+$
Geometric	ϑ_1	$\vartheta_1 + \vartheta_1^2$	$\vartheta_1 > 0$	log	$y \in \mathbb{N}_+$
Negative Binomial (I)	ϑ_1	$\vartheta_1 + \vartheta_2\vartheta_1^2$	$\vartheta_2,\ \vartheta_2 > 0$	log, log	$y \in \mathbb{N}_+$
Zero-Altered Logarithmic	$\frac{(1-\vartheta_2)\alpha\vartheta_1}{(1-\vartheta_1)}$	$\frac{(1-\vartheta_2)\alpha\vartheta_1(1-(1-\vartheta_2)\alpha\vartheta_1)}{(1-\vartheta_1)^2}$	$\vartheta_1,\ \vartheta_2 \in [0,1]$	logit, logit	$y \in \mathbb{N}_+$
Zero-Inflated Poisson	$(1-\vartheta_2)\vartheta_1$	$\vartheta_1(1-\vartheta_2)(1+\vartheta_1\vartheta_2)$	$\vartheta_1,\ \vartheta_2 \in [0,1]$	log, logit	$y \in \mathbb{N}_+$
Zero-Altered Negative Binomial	$c\vartheta_1$	$c\vartheta_1 + c\vartheta_1^2(1+\vartheta_2-c)$	$\vartheta_1, \vartheta_2 > 0,\ \vartheta_3 \in [0,1]$	log, log, logit	$y \in \mathbb{N}_+$
Zero-Inflated Negative Binomial	$(1-\vartheta_3)\vartheta_1$	$\vartheta_1(1-\vartheta_3) + \vartheta_1^2(1-\vartheta_3)(\vartheta_2+\vartheta_3)$	$\vartheta_1, \vartheta_2 > 0,\ \vartheta_3 \in [0,1]$	log, log, logit	$y \in \mathbb{N}_+$

Negative gradients of the implemented loss functions

Recall that the loss function corresponds to the negative log-likelihood, i.e. $\omega_i = -\ell_i$. The negative gradients of the loss with respect to the additive predictors are then the first partial derivatives of the log-likelihood with respect to the additive predictors, i.e. $-\partial\omega/\partial\eta_{ik}^{(\bullet)} = -\partial(-\ell_i)/\partial\eta_{ik}^{(\bullet)} = \partial\ell_i/\partial\eta_{ik}^{(\bullet)}$, with $\bullet \in \{1, 2, c\}$.

Bivariate binary responses The log-likelihood function for bivariate binary responses is given by:

$$\ell_i = y_{i1}y_{i2}\log(p_i^{11}) + y_{i1}(1-y_{i2})\log(p_i^{1(1)} - p_i^{11}) + \\ (1-y_{i1})y_{i2}\log(p_i^{1(2)} - p_i^{11}) + (1-y_{i1})(1-y_{i2})\log(1 - p_i^{1(1)} - p_i^{1(2)} + p_i^{11}).$$

where $p_i^{11} = C[\ p_i^{1(1)},\ p_i^{1(2)}; \vartheta_i^{(c)}\ ]$, $p_i^{1(1)} = P(Y_{i1} = 1; \vartheta_i^{(1)})$, and $p_i^{1(2)} = P(Y_{i2} = 1; \vartheta_i^{(2)})$. The negative gradients are then:

$$\frac{\partial\ell_i}{\partial\eta_i^{(1)}} = \left\{ \frac{y_{i1}y_{i2}}{p_i^{11}}\frac{\partial p_i^{11}}{\partial p_i^{1(1)}} + \frac{y_{i1}(1-y_{i2})}{p_i^{1(1)} - p_i^{11}}\left[1 - \frac{\partial p_i^{11}}{\partial p_i^{1(1)}}\right] - \right. \\ \left. \frac{(1-y_{i1})y_{i2}}{p_i^{1(2)} - p_i^{11}}\frac{\partial p_i^{11}}{\partial p_i^{1(1)}} - \frac{(1-y_{i1})(1-y_{i2})}{1 - p_i^{1(1)} - p_i^{1(2)} + p_i^{11}}\left[1 - \frac{\partial p_i^{11}}{\partial p_i^{1(1)}}\right] \right\} \frac{\partial p_i^{1(1)}}{\partial\eta_i^{(1)}},$$

$$\frac{\partial\ell_i}{\partial\eta_i^{(2)}} = \left\{ \frac{y_{i1}y_{i2}}{p_i^{11}}\frac{\partial p_i^{11}}{\partial p_i^{1(2)}} - \frac{y_{i1}(1-y_{i2})}{p_i^{1(1)} - p_i^{11}}\frac{\partial p_i^{11}}{\partial p_i^{1(2)}} + \right. \\ \left. \frac{(1-y_{i1})y_{i2}}{p_i^{1(2)} - p_i^{11}}\left[1 - \frac{\partial p_i^{11}}{\partial p_i^{1(2)}}\right] - \frac{(1-y_{i1})(1-y_{i2})}{1 - p_i^{1(1)} - p_i^{1(2)} + p_i^{11}}\left[1 - \frac{\partial p_i^{11}}{\partial p_i^{1(2)}}\right] \right\} \frac{\partial p_i^{1(2)}}{\partial\eta_i^{(2)}},$$

$$\frac{\partial\ell_i}{\partial\eta_i^{(c)}} = \left\{ \frac{y_{i1}y_{i2}}{p_i^{11}}\frac{\partial p_i^{11}}{\partial\vartheta_i^{(c)}} - \frac{y_{i1}(1-y_{i2})}{p_i^{1(1)} - p_i^{11}}\frac{\partial p_i^{11}}{\partial\vartheta_i^{(c)}} - \right. \\ \left. \frac{(1-y_{i1})y_{i2}}{p_i^{1(2)} - p_i^{11}}\frac{\partial p_i^{11}}{\partial\vartheta_i^{(c)}} + \frac{(1-y_{i1})(1-y_{i2})}{1 - p_i^{1(1)} - p_i^{1(2)} + p_i^{11}}\frac{\partial p_i^{11}}{\partial\vartheta_i^{(c)}} \right\} \frac{\partial\vartheta_i^{(c)}}{\partial\eta_i^{(c)}}.$$

Bivariate discrete responses The log-likelihood function for bivariate discrete responses is given by:

$$\begin{aligned}\ell_i &= \log\Big\{C[F_{1i},\ F_{2i};\ \vartheta_i^{(c)}]\ - C[F_{1i}-f_{1i},\ F_{2i};\ \vartheta_i^{(c)})] - \\ &\quad C[F_{1i},\ F_{2i}-f_{2i};\ \vartheta_i^{c}]\ + C[F_{1i}-f_{1i},\ F_{2i}-f_{2i};\ \vartheta_i^{c}]\Big\} \\ &= \log\Big\{\Delta_i\Big\},\end{aligned}$$

where the marginal CDFs and PDFs have been abbreviated as $F_{ji} = F_j(y_{ij};\boldsymbol{\vartheta}_i^{(j)})$ and $f_{ji} = f_j(y_{ij};\boldsymbol{\vartheta}_i^{(j)})$, $j = 1, 2$, in order to avoid clutter in the notation. The negative gradients are then:

$$\begin{aligned}\frac{\partial \ell_i}{\partial \eta_{ik}^{(1)}} &= \frac{1}{\Delta_i}\Bigg\{\left[\frac{\partial C[F_{1i},\ F_{2i};\ \vartheta_i^{(c)}]}{\partial F_{1i}} - \frac{\partial C[F_{1i},\ F_{2i}-f_{2i};\ \vartheta_i^{(c)}]}{\partial F_{1i}}\right]\frac{\partial F_{1i}}{\partial \eta_{ik}^{(1)}} + \\ &\quad \left[\frac{\partial C[F_{1i}-f_{1i},\ F_{2i}-f_{2i};\ \vartheta_i^{(c)}]}{\partial F_{1i}-f_{1i}} - \frac{\partial C[F_{1i}-f_{1i},\ F_{2i};\ \vartheta_i^{(c)}]}{\partial F_{1i}-f_{1i}}\right]\left[\frac{\partial F_{1i}}{\partial \eta_{ik}^{(1)}} - \frac{\partial f_{1i}}{\partial \eta_{ik}^{(1)}}\right]\Bigg\},\end{aligned}$$

$$\begin{aligned}\frac{\partial \ell_i}{\partial \eta_{ik}^{(2)}} &= \frac{1}{\Delta_i}\Bigg\{\left[\frac{\partial C[F_{1i},\ F_{2i};\ \vartheta_i^{(c)}]}{\partial F_{2i}} - \frac{\partial C[F_{1i}-f_{1i},\ F_{2i};\ \vartheta_i^{(c)}]}{\partial F_{2i}}\right]\frac{\partial F_{2i}}{\partial \eta_{ik}^{(2)}} + \\ &\quad \left[\frac{\partial C[F_{1i}-f_{1i},\ F_{2i}-f_{2i};\ \vartheta_i^{(c)}]}{\partial F_{2i}-f_{2i}} - \frac{\partial C[F_{1i},\ F_{2i}-f_{2i};\ \vartheta_i^{(c)}]}{\partial F_{2i}-f_{2i}}\right]\left(\frac{\partial F_{2i}}{\partial \eta_{ik}^{(2)}} - \frac{\partial f_{2i}}{\partial \eta_{ik}^{(2)}}\right)\Bigg\},\end{aligned}$$

$$\begin{aligned}\frac{\partial \ell_i}{\partial \eta_i^{(c)}} &= \frac{1}{\Delta_i}\Bigg\{\frac{\partial C[F_{1i},\ F_{2i};\ \vartheta_i^{(c)}]}{\partial \vartheta_i^{(c)}} - \frac{\partial C[F_{1i}-f_{1i},\ F_{2i};\ \vartheta_i^{(c)}]}{\partial \vartheta_i^{(c)}} - \\ &\quad \frac{\partial C[F_{1i},\ F_{2i}-f_{2i};\ \vartheta_i^{(c)}]}{\partial \vartheta_i^{(c)}} + \frac{\partial C[F_{1i}-f_{1i},\ F_{2i}-f_{2i};\ \vartheta_i^{(c)}]}{\partial \vartheta_i^{(c)}}\Bigg\}\frac{\partial \vartheta_i^{(c)}}{\partial \eta_i^{(c)}},\end{aligned}$$

where $\partial F_{\bullet i}/\partial \eta_{ik}^{(\bullet)} = (\partial F_{\bullet i}/\partial \vartheta_{ik}^{(\bullet)})(\partial \vartheta_{ik}^{(\bullet)}/\partial \eta_{ik}^{(\bullet)})$ and $\partial f_{\bullet i}/\partial \eta_{ik}^{(\bullet)} = (\partial f_{\bullet i}/\partial \vartheta_{ik}^{(\bullet)})(\partial \vartheta_{ik}^{(\bullet)}/\partial \eta_{ik}^{(\bullet)})$, with $\bullet \in \{1, 2\}$.

Bivariate mixed binary-continuous responses The log-likelihood function for mixed binary-continuous responses is the following:

$$\ell_i = (1 - y_{i1})\log\left\{\frac{\partial C[F_{1i},\ F_2;\vartheta_i^{(c)}]}{\partial F_{2i}}\right\}\ +\ y_{i1}\log\left\{1 - \frac{\partial C[F_{1i},\ F_{2i};\vartheta_i^{(c)}]}{\partial F_{2i}}\right\} + \log\left[f_{2i}\right],$$

where the marginal CDFs and the marginal PDF of the second margin have been abbreviated as $F_{1i} = F_1(0;\vartheta_i^{(1)})$, $F_{2i} = F_2(y_{i2};\boldsymbol{\vartheta}_i^{(2)})$, and $f_{2i} = f_2(y_{i2};\boldsymbol{\vartheta}_i^{(2)})$, respectively. The negative

gradients are then:

$$\frac{\partial \ell_i}{\partial \eta_i^{(1)}} = \left\{ \frac{(1-y_{i1})}{\frac{\partial C[F_{1i},\ F_2; \vartheta_i^{(c)}]}{\partial F_{2i}}} - \frac{y_{i1}}{1 - \frac{\partial C[F_{1i},\ F_{2i}; \vartheta_i^{(c)}]}{\partial F_{2i}}} \right\} \frac{\partial^2 C[F_{1i},\ F_2; \vartheta_i^{(c)}]}{\partial F_{1i}\ \partial F_{2i}} \frac{\partial F_{1i}}{\partial \eta_i^{(1)}},$$

$$\frac{\partial \ell_i}{\partial \eta_{ik}^{(2)}} = \left\{ \frac{(1-y_{i1})}{\frac{\partial C[F_{1i},\ F_2; \vartheta_i^{(c)}]}{\partial F_{2i}}} - \frac{y_{i1}}{1 - \frac{\partial C[F_{1i},\ F_{2i}; \vartheta_i^{(c)}]}{\partial F_{2i}}} \right\} \frac{\partial^2 C[F_{1i},\ F_2; \vartheta_i^{(c)}]}{\partial F_{2i}^2} \frac{\partial F_{2i}}{\partial \eta_{ik}^{(2)}} + \frac{1}{f_{2i}} \frac{\partial f_{2i}}{\partial \eta_{ik}^{(2)}},$$

$$\frac{\partial \ell_i}{\partial \eta_i^{(c)}} = \left\{ \frac{(1-y_{i1})}{\frac{\partial C[F_{1i},\ F_2; \vartheta_i^{(c)}]}{\partial F_{2i}}} - \frac{y_{i1}}{1 - \frac{\partial C[F_{1i},\ F_{2i}; \vartheta_i^{(c)}]}{\partial F_{2i}}} \right\} \frac{\partial C[F_{1i},\ F_2; \vartheta_i^{(c)}]}{\partial \vartheta_i^{(c)}} \frac{\partial \vartheta_i^{(c)}}{\partial \eta_i^{(c)}},$$

where $\partial F_{2i}/\partial \eta_{ik}^{(2)} = (\partial F_{2i}/\partial \vartheta_{ik}^{(2)})(\partial \vartheta_{ik}^{(2)}/\partial \eta_{ik}^{(2)})$ and $\partial f_{2i}/\partial \eta_{ik}^{(2)} = (\partial f_{2i}/\partial \vartheta_{ik}^{(2)})(\partial \vartheta_{ik}^{(2)}/\partial \eta_{ik}^{(2)})$.

Part B

In this supplement we provide further details and results of the simulation studies conducted for the bivariate binary (Subsection B1), bivariate discrete (Subsection B2), and bivariate mixed binary-continuous responses (Subsection B3), as well as copula selection (Subsection B4). In the simulation studies we use multivariate proper scoring rules to evaluate the fit of the copula and univariate models. Specifically, we consider the negative log-likelihood (log-score) and the energy score, with the latter being defined as follows. Let $\boldsymbol{y} = (y_1, \ldots, y_d)^\top \in \mathbb{R}^d$ be a new observation with unknown distribution F_Y, and let $\hat{F}_Y$ be a forecast distribution for F_Y. The energy score is then given by

$$ES(F, \boldsymbol{y}) = \frac{1}{n}\sum_{i=1}^{n} ||\boldsymbol{X}_i - \boldsymbol{y}|| - \frac{1}{2n^2}\sum_{i=1}^{n}\sum_{j=1}^{n} ||\boldsymbol{X}_i - \boldsymbol{X}_j||,$$

where $||\cdot||$ denotes the Euclidean norm on $\mathbb{R}^d$ and $\boldsymbol{X}_1, \ldots, \boldsymbol{X}_n$ are iid copies with distribution $\hat{F}_Y$, for $\boldsymbol{X}_i = (X_{i1}, X_{i2}, \ldots, X_{id},) \in \mathbb{R}^d$, $i = 1, \ldots, n$ (Gneiting et al., 2008).

B1 Bivariate binary responses

Data generation We consider three data generating processes (DGPs) with increasing number of noise variables. Specifically, we generate $p_1 = 10$, $p_2 = 100$ and lastly $p_3 = 1000$ covariates, of which only six have are truly informative in one or several of the distribution parameters. These configurations lead to 50% ($p1$), 5% ($p2$) and 0.5% ($p3$) of the covariates being informative, respectively. The bivariate distribution of the binary components is created using a Gaussian copula with varying correlation between the margins. On average, the dependence between the margins of the synthetic data in terms of Kendall's τ lies within $[-0.993;\ 0.993]$, i.e. it ranges between very strong negative to very strong positive dependence. We generate the first margin from a probit model and the second margin from a cloglog model. Thus, the model has $K = 3$ distribution parameters and the DGP with p_3 covariates represents a high-dimensional setting with $p_3 \gg n$, resulting in effectively $p_3 \times K = 3000$ covariates since we fit all regressors to each distribution parameter. For this scenario we only consider DGPs with linear effects of the covariates, which reflect the data analysed in Section 4.1. Following Strömer et al. (2023), we generate the p_q, $q = 1, 2, 3$ covariates by sampling from a multivariate Gaussian distribution with Toeplitz covariance structure of the form $\Sigma_{ij} = \rho^{|i-j|}$ for $1 \leq i, j \leq p_q$, with $\rho = 0.5$ denoting the correlation between consecutive covariates x_j and x_{j+1}. We consider the following linear predictors

$$\begin{aligned}
\Phi^{-1}\left(p_i^{1(1)}\right) = \eta_{i1}^{(1)} &= -1x_{i2} + 0.5x_{i3} + 1x_{i4} - 0.5x_{i6},\\
\log\left(\left(-\log(1 - p_i^{1(2)})\right)\right) = \eta_{i1}^{(2)} &= 0.5x_{i1} - 1x_{i2} + 0.75x_{i3},\\
\tanh\left(\vartheta_i^{(c)}\right)^{-1} = \eta_i^{(c)} &= 0.5x_{i2} - 1.5x_{i3} + 1.5x_{i4}.
\end{aligned}$$

Results Table B2, Column (1) summarizes the performance scores. Overall, the copula model exhibits a better performance in terms of the negative log-likelihood (log-score) as well as the energy score, indicating a better fit of the bivariate distribution. In terms of univariate scores (Brier score and AUC), both the univariate and copula models show similar results, with the copula model outperforming the univariate model at predicting the second margin in the high-dimensional setting ($p_3 = 1000$). The selection rates of informative and non-informative covariates in each of the distribution parameters are given in Table B3, Column (1). These are the percentage of instances in which the informative and non-informative variables entered the model's additive predictors, respectively. Overall, the selection rates rates of non-informative covariates in the distribution parameters belonging to the marginal distributions are slightly higher as compared to corresponding rates obtained from separate univariate models. This is somewhat expected given the increased model complexity. On the other hand, the differences decrease considerably as the number of non-informative covariates increases. The selection rates of non-informative covariates in the dependence parameter $\vartheta^{(c)}$ are considerably lower than that of effects in the parameters of the margins, whereas the selection rates of informative variables are slightly lower than 100%. This suggests that the shrinkage is strongest in the dependence parameter and the chosen criterion to determine $\mathtt{m}_{\mathtt{stop}}$ slightly underfits the effects in the dependence. However, both the copula and univariate models correctly select the informative covariates in all margins. Figure B1(a) depicts the estimated coefficients with each row corresponding to $p_1 = 10$, $p_2 = 100$, $p_3 = 1000$, respectively. In low-dimensional settings (p_1), the copula model matches the univariate models in producing accurate estimates of all linear coefficients in the marginal distributions. In high-dimensional settings (p_3), the estimated coefficients corresponding to the margins exhibit similar performance as in settings with p_1 and p_2.

B2 Bivariate discrete responses

Data generation We consider two DGPs for bivariate count responses each including $p = 10$ covariates. In the first DGP, the covariates have a strictly linear effect on the distribution parameters, whereas in the second DGP we consider non-linear effects. These configurations lead to 60% and 50% of the covariates being informative in the linear and non-linear DGPs, respectively. The bivariate discrete distribution is constructed using a combination of a Zero-Altered Logarithmic distribution (ZALG, margin 1) with two parameters, and a Zero-Inflated Negative Binomial Type I distribution (ZINBI, margin 2), which has three parameters. The marginal distributions and number of covariates are chosen to resemble the data studied in Section 4.2. The components are linked through a Joe copula, which allows to model positive dependence as well as upper tail dependence between the margins. The additive predictor of the dependence parameter $\vartheta_i^{(c)}$ covers Kendall's τ values within [0.275; 0.899], ranging from moderate to very strong positive dependence between Y_1 and Y_2. The covariates are sampled from independent univariate Uniform distributions with support between 0 and 1,

i.e. $X_r \sim U[0,1]$, $\forall r = 1, \dots, 10$. Overall, the bivariate distribution consists of six parameters with the following additive predictors

Linear DGP:

$$\log\left(\frac{\vartheta_{i1}^{(1)}}{1-\vartheta_{i1}^{(1)}}\right) = -1x_{i1} + 1x_{i3},$$
$$\log\left(\frac{\vartheta_{i2}^{(1)}}{1-\vartheta_{i2}^{(1)}}\right) = +1x_{i4} + 1x_{i5} - 2x_{i8},$$
$$\log\left(\vartheta_{i1}^{(2)}\right) = +1.5x_{i1} - 1.5x_{i2},$$
$$\log\left(\vartheta_{i2}^{(2)}\right) = -0.75x_{i2} + 1x_{i4},$$
$$\log\left(\frac{\vartheta_{i3}^{(2)}}{1-\vartheta_{i3}^{(2)}}\right) = -0.75x_{i2} + 1x_{i3},$$
$$\log\left(\vartheta_{i}^{(c)} - 1\right) = -0.5x_{i2} + 1.5x_{i3} + 1.5x_{i5},$$

Non-linear DGP:

$$\log\left(\frac{\vartheta_{i1}^{(1)}}{1-\vartheta_{i1}^{(1)}}\right) = \frac{1}{2}\left(x_{i1}^{3/2} - 2\cos(3x_{i1})\right),$$
$$\log\left(\frac{\vartheta_{i2}^{(1)}}{1-\vartheta_{i2}^{(1)}}\right) = -80\left(x_{i3}^{3/2} - x_{i3}^{4/3}\right),$$
$$\log\left(\vartheta_{i1}^{(2)}\right) = -0.7\exp\left(x_{i2}^{2}\right) + \exp\left(x_{i2}^{0.4}\right),$$
$$\log\left(\vartheta_{i2}^{(2)}\right) = 3 - 1.5\left(1.5\cos(2x_{i5}) + 3\tanh(x_{i5})\right),$$
$$\log\left(\frac{\vartheta_{i3}^{(2)}}{1-\vartheta_{i3}^{(2)}}\right) = -3 - 0.7\left(\sin(x_{i1}) - \exp(x_{i1})^2\right),$$
$$\log\left(\vartheta_{i}^{(c)} - 1\right) = 2\sin(4x_{i4}).$$

Note that in case of the linear DGP, seven out of the ten covariates have a non-zero effect on the distribution parameters with five of those overlapping and one having an effect uniquely on one parameter. In the non-linear DGP, six covariates are informative and once again there is some overlap in the informative covariates across parameters.

Results The performance metrics for the bivariate count response scenario are summarized in Table B2, Column (2). In terms of log- and energy scores, our proposed copula approach outperforms the univariate models considerably. The copula also leads to a smaller MSEP for predicting Y_2, whereas the univariate model for Y_1 outperforms the copula in terms of MSEP. The selection rates in Table B3, Column (2) demonstrate that the copula model selects more non-informative variables in the first margin, as well as in the first parameter of margin 2 compared to the univariate models in the linear DGP. However, the selection rates of informative covariates from the univariate models are considerably lower in the other two parameters of margin 2 compared to those of our copula model. These once again point out that the dependence parameter experiences the strongest shrinkage of the covariate effects. In the non-linear DGP, the selection rates of non-informative covariates are similar for the copula and univariate models in the first margin. In contrast, in the second margin the copula model tends to select too many non-informative covariates in the first parameter of margin 2. Concerning the linear effects, our approach performs well given the overlap between covariate effects and distribution parameters. The shrinkage on the estimated coefficients corresponding to the dependence parameter exhibits a very similar behaviour to that observed in the bivariate binary scenario with $p_1 = 10$. The univariate models tend to underestimate the covariate effects in the second parameter of margin 1 and the third parameter of margin 2. Furthermore, the univariate models display a slightly higher variance in the estimated coefficients compared to those derived from the copula models. This observation is supported by Figure B2(b), which

indicates selection rates of informative covariates in the non-linear DGP are consistently at 100% across all models.

B3 Bivariate mixed responses

Data generation For the mixed binary-continuous response scenario we generate the binary margin using a probit model, whereas the continuous margin follows a heteroskedastic Gaussian distribution. The components are linked through a Clayton copula rotated by 270°, which supports dependence between very high values of Y_1 and low values of Y_2. The choice of margins and copula is based on the data on children undernutrition analysed in Subsection 4.3. A total of $p = 10$ covariates are obtained from independent univariate Uniform distributions between 0 and 1, i.e. $X_r \sim U[0,1]$, $\forall r = 1, \dots, 10$. Once again we study both a DGP with only linear effects and another with non-linear effects of the covariates. In these configurations 50% (linear DGP) and 30% (non-linear DGP) of the covariates are informative. The bivariate distribution features four parameters with following additive predictors

Linear DGP:

$$\begin{aligned}
\Phi^{-1}\left(\vartheta_{i1}^{(1)}\right) &= 1.5x_{i2} - 1x_{i3} + 1.5x_{i4},\\
\vartheta_{i1}^{(2)} &= 0.5x_{i2} + 1.5x_{i3},\\
\log\left(\vartheta_{i2}^{(2)}\right) &= 1x_{i5},\\
\log\left(-\vartheta_{i}^{(c)}\right) &= 1.5x_{i5} - 1.5x_{i6},
\end{aligned}$$

Non-linear DGP:

$$\begin{aligned}
\Phi^{-1}\left(\vartheta_{i1}^{(1)}\right) &= \frac{1}{2}\left(x_{i1}^{3/2} - 2\cos(3x_{i1})\right),\\
\vartheta_{i1}^{(2)} &= -0.7\exp\left(x_{i1}^{2}\right) + \exp\left(x_{i1}^{0.4}\right),\\
\log\left(\vartheta_{i2}^{(2)}\right) &= -0.5 + \cos(2x_{i2})\\
\log\left(-\vartheta_{i}^{(c)}\right) &= -1 + 3\sin(4x_{i3}).
\end{aligned}$$

In the linear DGP, only five covariates have a non-zero effect on the distribution parameters and once again there is some overlap between informative covariates and distribution parameters. In the non-linear DGP, there are four informative covariates with some overlap between parameters and informative covariates.

Results From Table B2, Column (3) it can be observed that the copula model outperforms the univariate models in terms of the log and energy scores, with the difference in these two models becoming more apparent in the non-linear DGP. In the linear DGP, the copula model performs better than the univariate models in terms of the log-score but slightly worse in terms of the energy score, albeit the difference in energy scores is 0.001 and the univariate models exhibit a much larger standard deviation in the aforementioned score. Regarding the univariate scores, once again both copula and univariate models exhibit similar performance. In the non-linear DGP, both models are able to recover the true non-linear effects of the informative covariates (see Figure B2(c)). In the linear DGP a slight improvement in efficiency can be observed from the copula model. We remark that the boosted copula model once again exhibits a higher degree of shrinkage of the effects present in the copula parameter, as indicated by the selection rates in Table B3, Column (3). Similar to the other two response

scenarios, both copula and univariate models effectively identify the informative covariates across all distribution parameters of the margins. In the non-linear DGP, the copula model demonstrates a tendency to exhibit higher selection rates of non-informative variables within the continuous margin, while remaining competitive in the binary margin.

B4 Copula selection via the out-of-sample negative log-likelihood

We investigate the performance of the the out-of-sample negative log-likelihood to select the correct copula function under different a growing number of candidate covariates that enter the model. We generate bivariate binary data from a Gaussian copula with varying dependence using the configuration from Subsection B1. Hence the predictors that generate the dependent bivariate binary responses are given by:

$$\begin{aligned} \Phi^{-1}\left(p_i^{1(1)}\right) = \eta_{i1}^{(1)} &= -1x_{i2} + 0.5x_{i3} + 1x_{i4} - 0.5x_{i6}, \\ \log\left(\left(-\log(1-p_i^{1(2)}\right)\right) = \eta_{i1}^{(2)} &= 0.5x_{i1} - 1x_{i2} + 0.75x_{i3}, \\ \tanh\left(\vartheta_i^{(c)}\right)^{-1} = \eta_i^{(c)} &= 0.5x_{i2} - 1.5x_{i3} + 1.5x_{i4}. \end{aligned}$$

We then fit the following candidate distributions to the data:

1. Independent Bernoulli margins.
2. Gaussian copula with Bernoulli margins (correct specification).
3. Clayton copula with Bernoulli margins.
4. Clayton copula rotated by 90° with Bernoulli margins.
5. Clayton copula rotated by 180° with Bernoulli margins.
6. Gumbel copula with Bernoulli margins.

The link functions of the margins are correctly specified, and only the copula function is subject to misspecification. We consider $p_1 = 10$, $p_2 = 100$, and $p_3 = 1000$ covariates in the data. Similar to Subsection B1, 50% ($p1$), 5% ($p2$) and 0.5% ($p3$) of the covariates are informative in the respective cases. After finding the optimal number of fitting iteration of each boosting model as described in Algorithm 1, the out-of-sample negative log-likelihood is computed on a test set of size $n_{test} = 1000$ observations.

Results Table B1 shows the values of the out-of-sample negative log-likelihood for all candidate models and levels of sparsity. Overall, the correct copula (column 2, Gaussian) performs best.

Table B1: Simulation study. Out-of-sample negative log-likelihood (standard errors shown in parentheses) of fits from six candidate distributions (columns) to the true generating process that employs a Gaussian copula. The rows correspond to the different levels of sparsity. Lower values indicate better perfomance.

	True copula: Gaussian											
	Independence		Gaussian		Clayton 0°		Clayton 90°		Clayton 180°		Gumbel 0°	
$p_1 = 10$	974.33	(23.03)	**881.83**	(22.00)	916.67	(23.64)	1221.81	(24.70)	1324.32	(13.77)	921.96	(23.65)
$p_2 = 100$	985.59	(21.28)	**897.97**	(21.15)	953.82	(24.72)	1239.07	(20.92)	1327.56	(13.04)	961.56	(24.26)
$p_3 = 1000$	998.88	(19.70)	**915.30**	(20.17)	987.77	(22.62)	1256.22	(22.84)	1329.93	(13.93)	1004.60	(22.61)

Table B2: Simulation study. Performance metrics for the simulation studies for the copula (C) and univariate models (U), $\star$ identifies the non-linear DGP. Values are mean scores from 200 independent replicates (each evaluated on the test dataset), whereas parentheses show the respective standard deviations.

			(1)		(2)	(3)
Score	Model		Bivariate binary		Bivariate count	Mixed
		$p_1 = 10$	$p_2 = 100$	$p_3 = 1000$	$p = 10$	$p = 10$
Log	C	887.186 (24.103)	914.295 (22.364)	954.294 (21.120)	1579.591 (48.609)	1990.636 (28.791)
	U	956.639 (24.892)	971.851 (22.868)	998.185 (20.904)	1961.514 (64.262)	2011.168 (27.048)
	$C\star$	-	-	-	2442.732 (57.129)	1755.638 (31.690)
	$U\star$	-	-	-	2822.878 (64.924)	1911.316 (31.211)
Energy	C	0.279 (0.008)	0.284 (0.007)	0.293 (0.007)	0.725 (0.041)	0.687 (0.013)
	U	0.285 (0.008)	0.290 (0.007)	0.298 (0.007)	0.741 (0.041)	0.710 (0.466)
	$C\star$	-	-	-	1.581 (0.071)	0.673 (0.015)
	$U\star$	-	-	.	1.601 (0.069)	0.679 (0.015)
Brier (Y_1)	C	0.142 (0.006)	0.144 (0.007)	0.148 (0.006)	-	0.200 (0.006)
	U	0.142 (0.006)	0.144 (0.007)	0.148 (0.006)	-	0.199 (0.006)
	$C\star$	-	-	-	-	0.174 (0.006)
	$U\star$	-	-	-	-	0.174 (0.006)
Brier (Y_2)	C	0.177 (0.006)	0.180 (0.006)	0.185 (0.006)	-	-
	U	0.177 (0.006)	0.180 (0.006)	0.185 (0.005)	-	-
	$C\star$	-	-	-	-	-
	$U\star$	-	-	-	-	-
AUC (Y_1)	C	0.879 (0.010)	0.876 (0.012)	0.870 (0.012)	-	0.760 (0.015)
	U	0.879 (0.010)	0.876 (0.012)	0.871 (0.012)	-	0.760 (0.015)
	$C\star$	-	-	-	-	0.816 (0.012)
	$U\star$	-	-	-	-	0.816 (0.012)
AUC (Y_2)	C	0.796 (0.015)	0.791 (0.014)	0.776 (0.015)	-	-
	U	0.796 (0.015)	0.791 (0.014)	0.780 (0.015)	-	-
	$C\star$	-	-	-	-	-
	$U\star$	-	-	-	-	-
MSEP (Y_1)	C	-	-	-	1.084 (0.133)	-
	U	-	-	-	1.073 (0.132)	-
	$C\star$	-	-	-	1.563 (0.302)	-
	$U\star$	-	-	-	1.556 (0.302)	-
MSEP (Y_2)	C	-	-	-	2.504 (0.557)	1.190 (0.058)
	U	-	-	-	2.412 (0.581)	1.188 (0.058)
	$C\star$	-	-	-	10.799 (1.150)	1.293 (0.072)
	$U\star$	-	-	-	11.058 (1.221)	1.290 (0.072)
Copula			Gaussian		Joe	Rotated Clayton 270°
Kendall's τ range			$[-0.993;\ 0.993]$		$[0.275;\ 0.899]$	$[-0.787;\ -0.019]$

Gradients stabilised using L_2 norm, step-length $\texttt{s}_{\texttt{step}} = 0.1$. $n_{\text{train}} = 1000$, $n_{\text{test}} = 1000$, $n_{\texttt{mstop}} = 1500$.

Table B3: Simulation study. Selection rates (in %) of informative (x_{inf}) and non-informative ($x_{\text{n-inf}}$) covariates for the copula (C) and univariate models (U) for each distribution parameter, $\star$ denotes non-linear DGP. Values are averages over 200 independent datasets.

	(1)						(2)		(3)	
	Bivariate binary						Bivariate count		Binary & continuous	
	$p_1 = 10$		$p_2 = 100$		$p_3 = 1000$		$p = 10$		$p = 10$	
	x_{inf}	$x_{\text{n-inf}}$	x_{inf}	$x_{\text{n-inf}}$	x_{inf}	$x_{\text{n-inf}}$	x_{inf}	$x_{\text{n-inf}}$	x_{inf}	$x_{\text{n-inf}}$
**Linear DGP**										
Copula model (C)										
$\vartheta_1^{(1)}$	100	78.833	100	26.297	100	6.189	99	59.062	100	64.000
$\vartheta_2^{(1)}$	-	-	-	-	-	-	100	64.714	-	-
$\vartheta_1^{(2)}$	100	77.929	100	28.387	100	7.142	100	72.812	100	74.500
$\vartheta_2^{(2)}$	-	-	-	-	-	-	97	50.250	100	81.611
$\vartheta_3^{(2)}$	-	-	-	-	-	-	75.750	48.812	-	-
$\vartheta^{(c)}$	95.125	54.167	70.000	6.609	55.750	0.209	89.500	40.643	87.500	27.687
Univariate models (U)										
$\vartheta_1^{(1)}$	100	69.583	100	22.745	100	4.785	95.250	38.250	100	56.071
$\vartheta_2^{(1)}$	-	-	-	-	-	-	100	46.571	-	-
$\vartheta_1^{(2)}$	100	70.643	100	20.722	100	4.643	100	60	100	32.187
$\vartheta_2^{(2)}$	-	-	-	-	-	-	90.750	56.750	100	38.778
$\vartheta_3^{(2)}$	-	-	-	-	-	-	36	18.375	-	-
**Non-linear DGP**										
Copula model ($C\star$)										
$\vartheta_1^{(1)}$	-	-	-	-	-	-	100	29.944	100	33.111
$\vartheta_2^{(1)}$	-	-	-	-	-	-	100	44.444	-	-
$\vartheta_1^{(2)}$	-	-	-	-	-	-	100	99.500	100	83.000
$\vartheta_2^{(2)}$	-	-	-	-	-	-	100	34.278	100	83.611
$\vartheta_3^{(2)}$	-	-	-	-	-	-	100	4.889	-	-
$\vartheta^{(c)}$	-	-	-	-	-	-	100	18.944	100	0.278
Univariate models ($U\star$)										
$\vartheta_1^{(1)}$	-	-	-	-	-	-	100	27.556	100	38.278
$\vartheta_2^{(1)}$	-	-	-	-	-	-	100	22.222	-	-
$\vartheta_1^{(2)}$	-	-	-	-	-	-	99.500	59.778	100	25.722
$\vartheta_2^{(2)}$	-	-	-	-	-	-	98.500	53.278	100	36.611
$\vartheta_3^{(2)}$	-	-	-	-	-	-	100	8.889	-	-

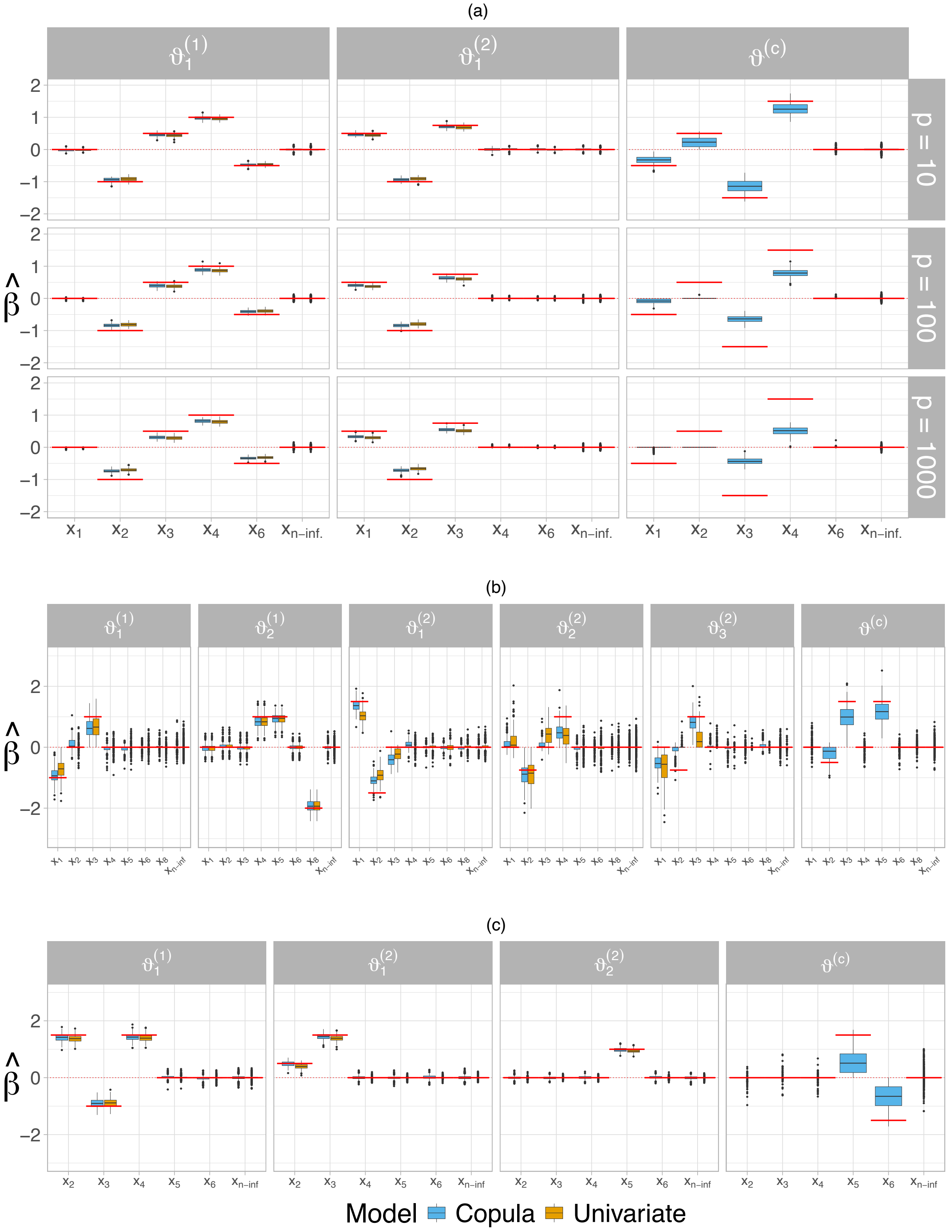

Figure B1: Simulation study. Estimated coefficients of informative covariates $x_1, \ldots, x_6$ and non-informative ($x_{\text{n-inf.}}$) covariates from copula and univariate models across distribution parameters for linear DGPs of Simulation B1 (a, Gaussian copula), B2 (b, Joe copula), B3 (c, rotated Clayton copula by 270°). Results obtained using 200 independent datasets.

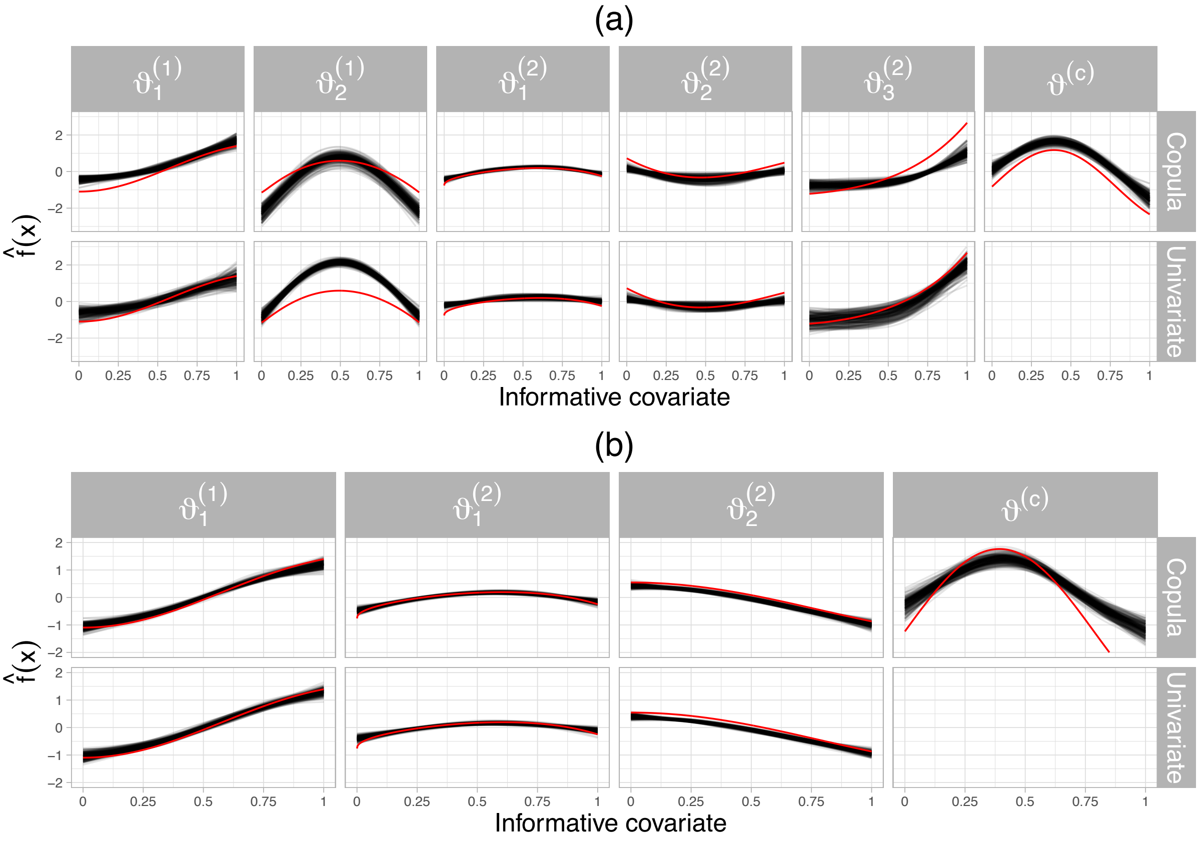

Figure B2: Simulation study. Estimated effects of the informative covariates from copula and univariate models across distribution parameters for non-linear DGPs of Simulation B2 (a, Joe copula) and B3 (b, rotated Clayton copula by 270°). Red lines indicate true effects. Results obtained using 200 independent datasets. The x-axis shows the corresponding covariate x_j and the y-axis the functional estimates $\hat{f}_j(x_j)$.

Part C

C1 Supporting results of Application 4.2

Table C1: Application 4.2. Out-of-sample negative log-likelihoods of the candidate discrete marginal distributions evaluated on $n_{test} = 778$ observations.

Marginal distribution	Negative log-likelihood Margin 1 (`doctorco`)	Margin 2 (`prescrib`)
Poisson	537.250	960.914
Geometric	523.207	906.440
Negative Binomial (I)	527.338	900.263
Zero-Altered Logarithmic	**522.664**	912.034
Zero-Altered Negative Binomial	524.863	898.757
Zero-Inflated Poisson	531.583	911.166
Zero-Inflated Negative Binomial	528.512	**895.975**

Table C2: Application 4.2. Out-of-sample negative log-likelihoods of the candidate copula functions and the bivariate Poisson distribution evaluated on $n_{test} = 778$ observations.

Copula	Negative log-likelihood
Independence	1418.639
Gaussian	1394.516
Clayton	**1392.381**
Gumbel	1406.620
Joe	1412.192
Farlie-Gumbel-Morgenstern	1401.902
Ali-Mikhail-Haq	1401.150
Bivariate Poisson distribution	1468.074

Table C3: Application 4.2. Selected base-learners across distribution parameters of the joint bivariate distribution of `doctorco` (ZALG) and `prescrib` (ZINBI).

Base-learner	Type	ZALG $\vartheta_1^{(1)}$	ZALG $\vartheta_2^{(1)}$	ZINBI $\vartheta_1^{(2)}$	ZINBI $\vartheta_2^{(2)}$	ZINBI $\vartheta_3^{(2)}$	Clayton copula $\vartheta^{(c)}$
`gender`	Linear	✓	✓	✓	✓	✓	✓
`age`	Non-linear	✓	✓	✓	✓	✓	✓
`income`	Non-linear	✓	✓	✓	✓		
`age:income`	Interaction			✓			
`age:gender`	Interaction	✓	✓	✓	✓	✓	
`income:gender`	Interaction			✓			

C2 Description and summary statistics of variables featured in Application 4.3

Table C4: Variables of Section 4.3. Responses are fever (binary, row 1) and wasting (continuous, row 2); covariates entering the model non-linearly are child's age, breastfeeding and the mother's body-mass-index (rows 3–5); the binary covariate child's gender enters the model linearly (row 5) and the district in India (row 6) enters the model as a discrete spatial effect.

Variable	Description	Type	Mean (s.d.)
`fever`	Fever experienced within two weeks preceding survey interview	Binary	0.307 (0.461)
`wasting`	Low weight-for-height	Continuous	−79.144 (123.367)
`cage`	Age of the child in months	Continuous	17.255 (10.148)
`breastfeeding`	Months of breastfeeding	Continuous	14.076 (8.751)
`mbmi`	Mother's Body-Mass-Index	Continuous	19.783 (2.937)
`cgender`	Gender of the child (1 female, 0 male)	Binary	0.476
`distH`	District of residence	Factor	-
Number of districts: 438, $n = 24{,}286$.			

References

Gneiting, T., Stanberry, L. I., Grimit, E. P., Held, L., and Johnson, N. A. (2008). Assessing probabilistic forecasts of multivariate quantities, with an application to ensemble predictions of surface winds. *TEST*, 17(2):211–235.
URL: https://doi.org/10.1007/s11749-008-0114-x.

Rigby, R. A., Stasinopoulos, M. D., Heller, G. Z., and De Bastiani, F. (2019). *Distributions for modeling location, scale, and shape: Using GAMLSS in R*. Chapman and Hall/CRC, New York.
URL: https://doi.org/10.1201/9780429298547.

Strömer, A., Klein, N., Staerk, C., Klinkhammer, H., and Mayr, A. (2023). Boosting multivariate structured additive distributional regression models. *Statistics in Medicine*, 42(11):1779–1801.
URL: https://doi.org/10.1002/sim.9699.
